# Sodium butyrate attenuates microglia-mediated neuroinflammation by modulating the TLR4/MyD88/NF-κB pathway and microbiome-gut-brain axis in cardiac arrest mice

**DOI:** 10.1186/s13041-025-01179-w

**Published:** 2025-02-17

**Authors:** Jianfei Sun, Liping Lu, Yingtao Lian, Song Xu, Ying Zhu, Yanping Wu, Qianhui Lin, Jing Hou, Yinping Li, Zhui Yu

**Affiliations:** 1 Department of Critical Care Medicine, Renmin Hospital of Wuhan University, No. 99 ZhangZhidong Road, Wuhan, 430060 Hubei China; 2 Central Laboratory, Renmin Hospital of Wuhan University, Wuhan, 430060 China; 3 Department of Anesthesiology, Renmin Hospital of Wuhan University, Wuhan, 430060 China; 4 Department of Pathophysiology, Hubei Province Key Laboratory of Allergy and Immunology, Taikang Medical School (School of Basic Medical Sciences), Wuhan University, Wuhan, 430060 China

**Keywords:** Cardiac arrest, Microglia, Sodium butyrate, TLR4/MyD88/NF-κB pathway, Neuroinflammation, Gut microbiota

## Abstract

Cardiac arrest (CA) is one of the most common illnesses worldwide. Post-CA brain injury (PCABI) is a major cause of death and poor recovery in CA patients and the current CA treatments are not very effective. The microbiome-gut-brain axis has been found to significantly affect brain ischemia injury. Furthermore, in ischemic stroke patients, short-chain fatty acids (SCFA), especially sodium butyrate (SB), have been observed to promote neuroprotective effects by modulating inflammatory response and microglial polarization in the cortex. However, the precise mechanism of SB on CA-induced injury remains elusive. Therefore, this research study established an oxygen–glucose deprivation and reoxygenation (OGD/R) model using BV-2 microglial and HT22 cells to simulate cerebral ischemia/reperfusion injury in vitro and a potassium chloride-induced CA mouse model to mimic CA in vivo. The data revealed that SB markedly improved neurological scores and reduced neuronal death and apoptosis. Moreover, it reduced M1 microglia and neuroinflammation in CA mice. In addition, SB increased intestinal integrity and alleviated systemic inflammation. The 16S rDNA sequencing analysis indicated that SB intervention mitigated CA-induced gut microbiota dysbiosis and SCFA depletion. It was also observed that CA mice’s brain and OGD/R-exposed BV2 cells had substantially increased levels of MyD88, phosphorylated NF-κB p65, and TLR4 proteins, which were reduced after SB treatment. In summary, this study revealed that SB can protect against cerebral ischemia–reperfusion injury by controlling microglia polarization and microbiome-gut-brain axis to inhibit brain inflammation via the TLR4/MyD88/NF-κB pathway.

**Supplementary Information:**

The online version contains supplementary material available at 10.1186/s13041-025-01179-w.

## Introduction

The 2023 American Heart Association has reported that after discharge, the survival rate of out-of-hospital cardiac arrest (OHCA) patients treated by Emergency Medical Services is 9.1%. Furthermore, despite significant improvements in cardiopulmonary resuscitation (CPR), patient’s survival with favorable functional status remains extremely low [1]. Moreover, patients who have been successfully resuscitated from cardiac arrest (CA) often experience post-CA syndrome (PCAS), such as brain injury, systemic ischemia–reperfusion response, and myocardial dysfunction [2]. Adrie, C., et al*.* [3] revealed that patients who undergo successful CPR after CA suffer from “systemic inflammatory response syndrome”, which may result in multiple organ dysfunction and even death. The brain is most susceptible to damage by anoxia and inflammation, and post-CA cerebral ischemia and hypoxia are the primary causes of mortality, long-term neurologic disability, and even death among survivors [4]. Hypothermia is predominantly employed as a therapeutic intervention for CA to enhance neurological recovery; however, it does not improve the mortality of OHCA patients compared to targeted normothermia [5]. Therefore, novel treatments are urgently required to combat brain damage in CA survivors, specifically targeted interventions against inflammation might be significant.

The central nervous system (CNS) predominantly includes microglia as immune cells in the parenchyma; therefore, they are the primary responders to any environmental perturbation [6]. Microglial activation and polarization have dual functions in neurological recovery and are crucial for injury and repair in ischemic stroke [7]. Furthermore, microglia can recognize harmful stimuli, polarize to classical activation (M1) state, and produce inflammatory cytokines including interleukin (IL) 1β, tumor necrosis factor-alpha (TNF-α), interferon-γ (IFN-γ), and IL-6. Microglia can also polarize to the M2 state, which expresses IL-10 and transforming growth factor-β (TGF-β) to protect the body from inflammation [8]. Therefore, inhibiting M2 to M1 polarization and promoting M2 polarization might be a potential therapeutic strategy. Several studies have indicated that CA/CPR causes severe neurological injury, which is related to substantially elevated pro-inflammatory cytokines, microglial activation, and neuroinflammation [9–11].

Microglia contains Toll-like receptors (TLRs), which modulate microglial activation and neurotoxicity [12]. The literature has indicated that TLR4 levels are upregulated during ischemia/reperfusion (I/R)-induced ischemic brain injury [13]. TLR4 activates the myeloid differentiation primary response protein 88 (MyD88) pathway in response to a particular stimulus, which interacts with tumor necrosis-associated factor 6 (TRAF6), promoting IκB phosphorylation and nuclear factor kappa light chain enhancer of activated B cells (NF-κB) transcription factors release, thus inducing pro-inflammatory cytokines, including TNFα, IL-6, and IL-1β [14]. It has been observed that in a murine CPR model after potassium-based CA, the TLR4/NF-κB signaling was activated and pro-inflammatory cytokines derived from microglia/monocytes were significantly elevated [15]. Furthermore, TLR4 genetically modified mice exhibited reduced brain injury and inflammation triggered by CA/CPR [16]. Wang, Z., et al [17]. revealed that TLR4/NLRP3 signaling modulates microglial activation and neuroinflammation in the brain following CA. These data are still limited to validate whether the signaling pathway of TLR4/MyD88/NF-κB promotes neuroprotection by regulating microglia during the early phase of CA-induced systemic I/R injury.

In the last 10 years, scientists have confirmed that in humans, resident microbiota are key modulators of host physiological mechanisms and a critical determinant of disease and health. The bidirectional interaction between the CNS and gut microbiota (GM) is termed the microbiome-gut-brain axis [18]. Several studies have indicated that GM dysbiosis is a predominant cause of different CNS diseases in humans and animals, including stroke [19–21], Parkinson’s disease [22], and Alzheimer’s disease [23]. The previous literature has confirmed that cardiac and cerebral injuries are a dominant cause of increased fatality and disability in CA patients; however, recently it was observed that intestinal injury is a key factor in the poor prognosis of CA survivors [24]. Furthermore, hypoxia and mesenteric ischemia induce bacterial translocation, thus aggravating post-resuscitation injuries [3]. These studies have highlighted the importance of GM dysbiosis in CA patients. In the microbiota-gut-brain axis, microglia are considered the target cells and might induce alterations in microbiome composition to modify brain function [25, 26]. However, the potential role of microglia in post-CA brain injury (PCABI) warrants comprehensive research. Therefore, PCABI might be treated by modulating gut microflora. Bacterial anaerobic fermentation of unabsorbed carbohydrates produces short-chain fatty acids (SCFA), which serve as mediators between the brain and GM and have been correlated with stroke severity and prognosis [27, 28]. Sodium butyrate (SB) is a SCFA, which can inhibit histone deacetylase and is implicated in ischemia stroke pathogenesis, specifically by converting the activated M1 (pro-inflammatory) microglial phenotype to M2 (anti-inflammatory) [29] and remodeling the GM [30]. However, studies on the potential effect of SB administration post-resuscitation intestinal and brain injury due to CA/CPR-induced systemic I/R injury, as well as the crosstalk between gut and brain are still lacking.

Therefore, this research study elucidated whether SB improves neurological outcomes and alleviates GM dysbiosis via the microbiota-gut-brain axis, utilizing an in vivo murine CA/CPR model and an in vitro co-culture system of BV2 microglia cells and HT22 neurons exposed to oxygen–glucose deprivation/reoxygenation (OGD/R). It was hypothesized that SB protects against brain injury by modulating the microglial phenotypic shift and the signaling pathway of TLR4/MyD88/NF-κB, which could mitigate the inflammatory response and alleviate CA/CPR-induced brain damage. Furthermore, the potential role of the microbiota-gut-axis in CA pathogenesis was also evaluated.

## Methods

### Animals

C57BL/6 mice (weight = 22.0—26.0 g, age = 8—10 weeks, male) were acquired from Hunan SJA Laboratory Animal Co., Ltd and kept in humidity and temperature controlled conditions with 10 h dark/14 h light cycle and ad libitum access to standard rodent chow and water. All animals were acclimatized for at least 1 week before subsequent experiments. All in vivo analyses were authorized by the Laboratory Animal Centre of Wuhan University (No. WDRM 20171204). All the mice were randomly categorized into 4 groups: Sham + Vehicle (saline), Sham + SB (300 mg/kg), CA/CPR + Vehicle, CA/CPR + SB.

### Cardiac arrest model

**Fig. 1:**
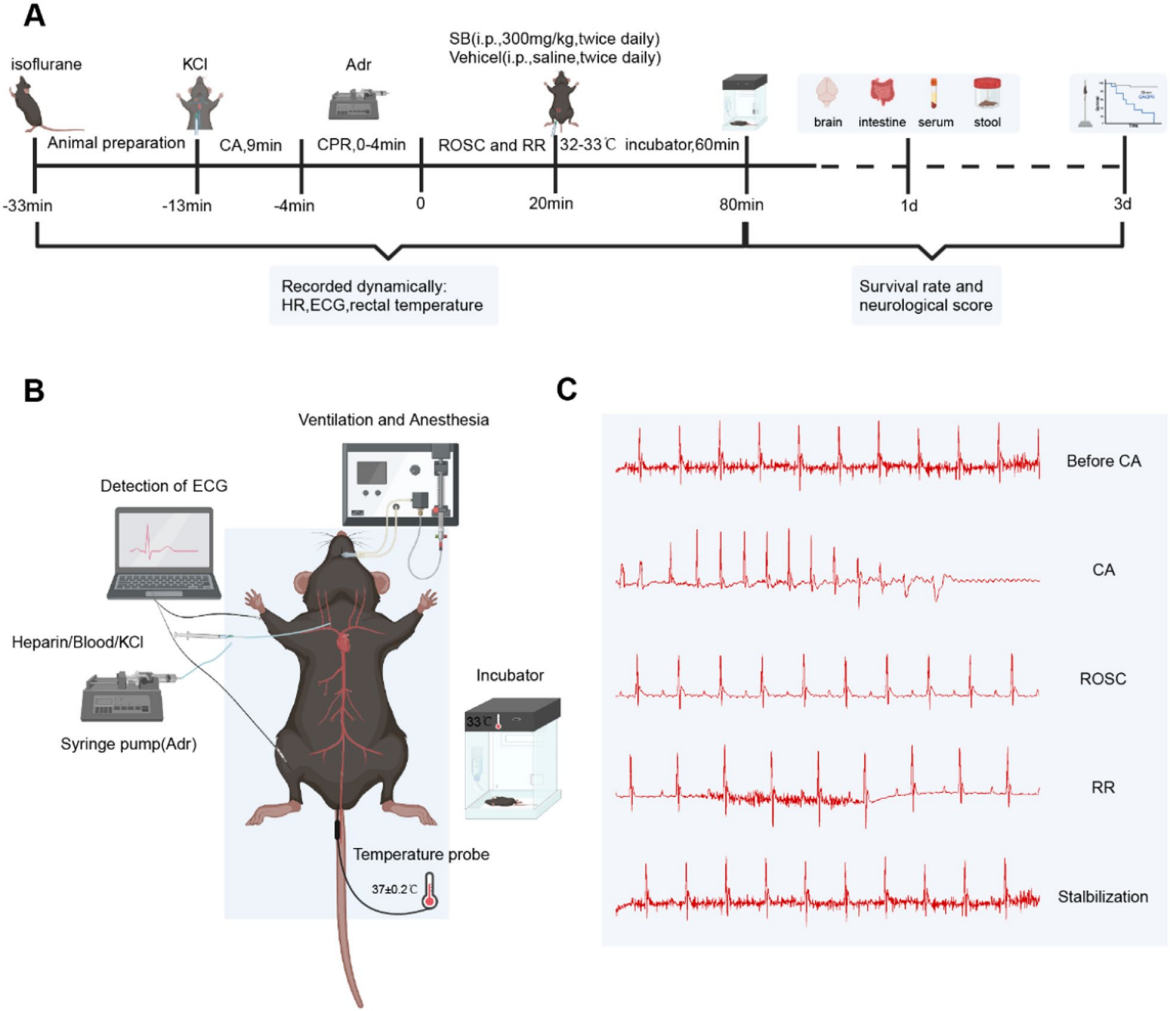
The animal modeling protocol. **A** Experimental flowchart. Briefly, the 9 min CA modeling protocol followed by CPR was carried out. After RR, vehicle (normal saline) or SB (300 mg/kg) in equal volume was intraperitoneally injected in mice. Further 24 h, the brain, gut, serum, and fecal samples were collected. The survival rate and neurological function were elucidated at 72 h post-CA/CPR or sham operation. **B** Schematic diagram of KCl-induced mouse CA model. **C** Representative waveforms of ECG during model establishment. *CPR* cardiopulmonary resuscitation, *KCl* potassium chloride, *Adr* adrenaline, *RR* return of respiration, *CA* cardiac arrest, *SB* sodium butyrate, *HR* heart rate, *ROSC* return of spontaneous circulation, *ECG* electrocardiogram

The CA model was generated by following a previously mentioned protocol [31, 32]. Briefly (Fig. 1), mice were anesthetized using 5% isoflurane for 2 min. To maintain, anesthesia at a concentration of 1% isoflurane, a 22 G cannula was intubated and connected to a mouse ventilator with the respiratory rate set at 142 breaths/min. The body temperature was assessed by inserting a temperature probe into the rectum. Subcutaneous needle electrodes monitored the electrocardiograms (ECGs) of mice throughout the experiment. For drug administration, a PE10 catheter pre-flushed with heparinized saline (0.9% NaCl) was administered into the right internal jugular vein and CA was induced by administering cold (4 °C) 0.5 M KCl (30 μL) through the jugular vein. The model was confirmed by asystole on the ECG monitor, absence of spontaneous respiration, and urinary incontinence. During CA, the ventilator and anesthesia were turned off and the body temperature was maintained at 37 ± 0.2 °C. The CPR was initiated after 9 min of inducing CA with rapid administration of 100 μL of adrenaline solution (16 μg/mL, 0.9% saline) followed by finger chest compressions at 300 beats/min and 100% oxygen ventilation. The return of electrical activity on the ECG monitor indicated the return of spontaneous circulation (ROSC). CPR was discontinued if no ROSC was observed for 4 min. After the mice were stabilized with spontaneous respiration, the surgical wounds were sutured and 500 μL saline was injected subcutaneously. The mice were then placed in a warm incubator at 33 °C for 1 h of observation, followed by transfer to separate cages with easy access to food and water. The sham mice received identical anesthesia and surgical exposure of the jugular vein, excluding CA and CPR.

### Drug administration

After the return of respiration (RR), SB (303,410-5G, Sigma-Aldrich; prepared in 0.9% saline) was administered intraperitoneally (i.p.) in mice twice daily. The administration dose was researched from previous literature [33].

### Neurological score

Neurological outcomes were evaluated 24, 48, and 72 h post-CA/CPR. The neurologic deficits were assessed using the 9-point scoring system [34]. The performance of mice on the horizontal bar, vertical screen, and rope was recorded. Each test was scored from 0 to 3 points, with a total score range of 0 – 9 (where 9 indicates normal function and 0 indicates severe impairment). Furthermore, for general neurological assessment, a 12-point scoring system [10] was employed, which mainly comprised the following 6 items: reaction to stimuli, corneal reflex, breathing, righting reflex, coordination, and movement. For each test, 0—2 points were given, and the total score ranged between 0 and 12 points, where 12 = normal function and 0 = severe damage. Daily survival rates and body weights were also monitored.

### Hematoxylin–eosin (H&E) and Nissl Staining

After anesthesia, the mice were intracardiacally perfused using 0.9% NaCl and subsequently preserved with 4% paraformaldehyde (PFA). Then, the brain, jejunum, ileum, and colon were harvested, preserved in 4% PFA for 24 h, dehydrated, and submerged in paraffin. Then, coronal sections of brain tissue and cross-sectional sections of intestinal tissue were prepared, stained with H&E, and visualized under a white light microscope. For Nissl staining, the slices were stained for 2—5 min with dye solution, dehydrated in graded ethanol, slightly differentiated with 0.1% glacial acetic acid, and then treated with xylene for transparency for 10 min before microscopic analysis.

### Immunohistochemistry (IHC) staining

For IHC staining, brain and intestinal tissue Sects. (5 µm) were paraffin-embedded, dewaxed to water, and subjected to antigen repair. Then, for blocking endogenous peroxidase, the sections were treated with 3% H_2_O_2_ for 25 min at ambient temperature away from light. Subsequently, the sections were blocked with 3% bovine serum albumin (BSA), treated overnight with primary Iba-1 (1:200) and occludin (1:500) antibodies at 4 °C, and probed with the corresponding secondary antibody at ambient temperature for 50 min. Then, freshly prepared diaminobenzidine was added to develop the color, and hematoxylin was used for restaining the nuclei. The sections were then dehydrated and sealed. Finally, and area of positive cells was calculated using ImageJ software.

### 16S rDNA sequencing of gut microbiota

After 24 h of CA/CPR, fresh feces were sampled from each group of mice using sterile tubes and stored at -80℃ for DNA isolation. Shanghai Majorbio Bio-Pharm Technology Co., Ltd. (China) performed the microbiome analysis. After genomic DNA extraction, specific primers containing barcodes were designed for the sequencing regions. QuantiFluor™ -ST Blue fluorescence quantification system (Promega Corporation) was employed for quantifying PCR products, which were then mixed based on each sample’s required sequencing volume. The single-strand DNA fragments were then sequenced and based on a 97% similarity threshold, all the high-quality reads were categorized into operational taxonomic units (OTUs). The community species composition of each sample was classified into various taxonomic levels from the phylum to the genus by the RDP classifier Bayesian algorithm.

### Gas chromatography-mass spectrometry (GC–MS) analysis of SCFA

Shanghai Majorbio Bio-Pharm Technology Co., Ltd. also analyzed the targeted metabolomics. Briefly, fresh stools were immediately frozen in liquid nitrogen. Then, 20 mg of the sample was taken in an Eppendorf (2 mL), mixed with 800 μL 0.5% phosphoric acid water [comprising 2-ethylbutyric acid (10 μg/mL) as an endogenous standard] to grind, centrifuged for 10 min at 13,000 g and 4 ℃, redissolved in 200 μL n-butanol solvent extraction and re-centrifuged at 13,000 g and 4 ℃ for 5 min before injecting into the GC–MS system. Agilent 8890B-7000D GC/MSD gas-mass spectrometer (Agilent Technologies Inc., CA, USA) was employed to quantify SCFA. Furthermore, SCFA concentrations in each sample were calculated by a standard curve.

### Cell culture and treatment

Mouse HT22 hippocampal neurons and BV2 microglial cells were acquired from Wuhan Pricella Biotechnology Co., Ltd. and cultured in high-glucose Dulbecco’s modified Eagle medium (DMEM) augmented with 1% penicillin/streptomycin and 10% fetal bovine serum at 37 °C in 95% air and 5% CO₂. Upon 60 – 70% confluency, cells were rinsed thrice with PBS and incubated in glucose- and serum-free DMEM for 2 h in 1% O₂, 94% N₂, and 5% CO₂ environment to induce ischemia-hypoxia, followed by re-incubated under standard culture conditions, with or without SB reperfusion for 24 h. Cells between passages 5 and 20 were used for experiments. These treated cells and their culture supernatants were sampled for immunofluorescence, western blot, ELISA, and flow cytometry analyses.

### Co‑culture of neurons and microglia

Upon 60 – 70% confluency, HT22 cells were cultured in glucose- and serum-free DMEM for 2 h in 1% O₂, 94% N₂, and 5% CO₂ environment. The medium was then replaced with conditioned medium and incubation was continued for 24 h. The conditioned medium was a mixture of the supernatant of BV2 cell culture medium, which was centrifuged at 1000 rpm for 5 min to remove cell debris, and DMEM (1:1). In total 4 experimental groups were established: Control (incubated with complete medium), Control + SB (incubated with complete medium + SB), OGD/R (incubated with OGD/R conditioned medium), OGD/R + SB (incubated with OGD/R + SB conditioned medium) groups.

### Cell counting kit-8 (CCK8) assay

Cellular viability was elucidated via the CCK8 assay (GK10001, GLPBIO). Briefly, both cell lines (1 × 10^4^/well) were plated in a 96-well plate for 24 h. Then, BV2 cells were treated with different SB concentrations (0—2.5 mM) for different OGD/R times to evaluate the optimal drug concentration for administration and the time of oxygen–glucose deprivation and reoxygenation. Whereas HT22 was subjected to OGD for 2 h and then the media were replaced with BV2-conditioned media for 24 h. Then, in each well, CCK-8 reagent (10 μL) mixed with DMEM (90 μL) was added at 37 °C for 2 h. The optical density (OD) was measured via a microplate reader (Thermo Fisher Scientific, Wilmington, DE, USA) at 450 nm.

### Immunofluorescence (IF) staining and TUNEL assay

Upon reaching 70% confluency, the cells were preserved for 20 min in 4% PFA, permeabilized for 20 min using Triton X-100 (0.5%) and sealed with BSA (3%) for 30 min. The specimens or cells were treated with primary antibodies: anti-CD86 (ab220188, Abcam, 1:200), anti-CD206 (60,143–1-Ig, Proteintech, 1:200), anti-NF-κB (#8242, Cell Signaling Technology, 1:500), and anti-Iba-1 (#17,198, Cell Signaling Technology, 1:300) at 4 °C overnight, washed and probed with the corresponding secondary antibody at ambient temperature for 1 h. Neuronal apoptosis was evaluated using TUNEL staining (Servicebio, Wuhan, China) as per the kit instructions. For nuclear staining, the samples were treated with Hoechst or DAPI solution for 10 min in darkness. The samples were imaged via a fluorescence microscope.

### Western blotting (WB)

The cells were subjected to RIPA lysis buffer augmented with the cocktail of protease inhibitors for 30 min on ice and then centrifuged at 12,000 × g for 15 min at 4 °C to collect the supernatant. Cerebral tissues were acquired from each group (n = 6 samples/group), homogenized using cold lysis buffer comprising protease inhibitors, and centrifuged for 15 min at 14,000 × g to acquire supernatant. The proteins in the supernatants were quantified via a BCA protein assay kit (P0010, Beyotime), then mixed with 5 × loading buffer (G2013, Servicebio) and heated for 5 min. Then, the proteins were isolated on 10—15% SDS-PAGE electrophoresis and transferred to the polyvinylidene fluoride (PVDF) membrane, which was sealed with 5% skim milk for 1 h, and treated with the following primary antibodies: NF-κB (#8242, Cell Signaling Technology, 1:1000), TLR4 (66,330–1-Ig, Proteintech, 1:1000), MyD88 (67,969–1-Ig, Proteintech, 1:1000), GAPDH (GB15002, Servicebio, 1:5000), p-NF-κB (#3033, Cell Signaling Technology,1:1000) and Iba-1 (17,198, Cell Signaling Technology, 1:1000) at 4 °C overnight. The membranes were then rinsed thrice, probed with appropriate secondary antibodies at ambient temperature for 1 h, and reacted with enhanced chemiluminescence (ECL) reagent (HY-K2005, MedChemExpress) to generate protein bands. The ImageJ software was employed to quantify proteins.

### Flow cytometry

BV2 cells (1 × 10^6^) were suspended in 100 μL PBS, treated with anti-mouse CD16/32 (101,302, BioLegend) to block Fc receptor binding, followed by treatment with APC anti-mouse CD86 monoclonal antibody (159,216, BioLegend) and PE anti-mouse CD206 monoclonal antibody (141,706, BioLegend) for 30 min in the dark. Then, the cells were rinsed with stream buffer and responded to 500 μL of stream buffer. For neuronal apoptosis, co-cultured HT22 cells were digested via 0.25% EDTA-free trypsin, rinsed with PBS twice, resuspended in 100 µL of binding buffer, and stained with a 1:1 mixture of PI (2.5 μL) and V-FITC (2.5 μL) (E-CK-A211, Elabscience) for 15 min under at ambient temperature without light. Lastly, cells were treated with 400 μL of Annexin V binding buffer. Microglial polarization and neuronal apoptosis were assessed via flow cytometry (CytoFlex, Beckman Coulter, USA).

### Enzyme-linked immunosorbent assay (ELISA)

The content of inflammatory factors including TNF-α (MU30030), IL-6 (MU30044), IL-10 (MU30055), and TGF-β (MU30574) (all from Bioswamp) was assessed via ELISA, per the kit’s protocols. The separated half of the lateral cortex was homogenized in 1 mL PBS. The blood and homogenates were centrifuged for 15 min at 12,000 × g to collect the serum and supernatant. BV2 cells were grown in 10 cm dishes and then subjected to OGD/R. The culture supernatants were centrifuged at 12,000 × g for 15 min and then collected for detection. The OD was measured via a microplate reader at 450 nm.

#### Statistical analysis

All the statistical assessments were carried out via GraphPad Prism software 8.0 and the values were depicted as mean ± standard error of the mean (SEM). The inter-group differences were assessed via the Kruskal–Wallis H test or one-way analysis of variance (ANOVA). Statistical significance was defined as ****p < *0.0001,* ***p < *0.001*, **p < *0.01*, and *p < *0.05*.

## Results

### SB has a protective effect on the brain after CA

**Fig. 2:**
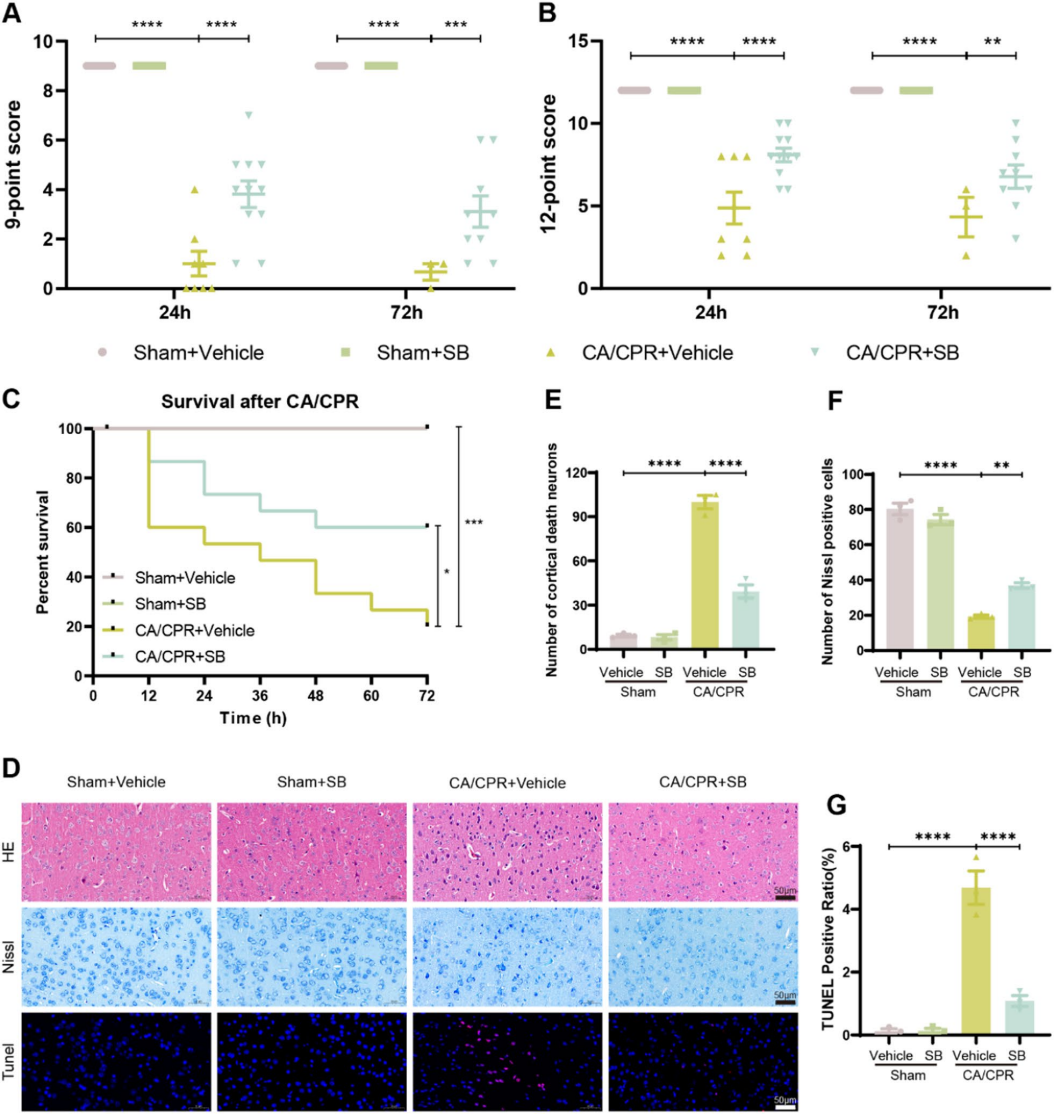
SB alleviated CA/CPR-induced neurological dysfunction and neuronal injury. After 24 and 72 h of CA/CPR, **A** 9-point score and **B** 12-point score were measured in four groups, respectively. **C** Kaplan–Meier curves of cumulative survival were generated 72 h post-CA/CPR for the different groups. (n = 15/group). **D** Representative HE, Nissl, Tunel staining images of cells 24 h post-ROSC. Scale bar: 50 µm (200 ×). **E** Counts of dead neurons. **F** Number of Nissl bodies per group. **G** The Tunel positive ratio. Data are depicted as mean ± SEM (n = 3/group). ****p < *0.0001,* ***p < *0.001*, **p < *0.01*, *p < *0.05*

After 3 days of CA/CPR, the neurological function and mortality were evaluated. It was observed that the CA/CPR group had markedly reduced scores relative to the sham group, irrespective of the 9- or 12-point scoring systems; however, this effect was reversed after SB treatment (Table 1, Fig. 2A, B). Furthermore, the survival rates at 24, 48, and 72 h were 53.3% (8 mice), 33.3% (5 mice), and 20% (3 mice) in the CA/CPR group, respectively. Whereas SB-treated mice survival rates were markedly enhanced during 3 days (Fig. 2C). To evaluate CA/CPR-induced histological injury, H&E and Nissl staining analyses were performed at 24 h post-resuscitation. The H&E staining results (Fig. 2D) suggested that the CA/CPR group had substantially increased dead neurons with shrunken profiles, karyopyknosis, intensely stained nuclei, and severe infiltration of inflammatory cells compared to the sham group (P < 0.0001). However, morphological injuries and reduced the number of dead neurons were substantially mitigated by SB treatment (P < 0.0001, Fig. 2E). Furthermore, Nissl staining indicated that CA/CPR mice had a reduced number of Nissl-positive neurons in the cortex (Sham + Vehicle vs CA/CPR + Vehicle, P < 0.0001), which was markedly enhanced after SB treatment (CA/CPR + Vehicle vs CA/CPR + SB, P = 0.002, Fig. 2F). TUNEL staining analysis was carried out to assess neuronal apoptosis in the cortex, which revealed robust cellular apoptosis in the CA/CPR mice cortex compared to the sham mice (P < 0.0001, Fig. 2D), whereas SB alleviated this effect (P < 0.0001, Fig. 2G). These results indicated that SB ameliorated CA/CPR-induced brain injury in mice.

**Table 1:** Scores of 9 and 12 were measured in the four groups at the 24-h and 72-h time points, respectively

12-point score											9-point score								
	Sham + Vehicle	Sham + SB	CA/CPR + Vehicle	CA/CPR + SB		Sham + Vehicle	Sham + SB	CA/CPR + Vehicle	CA/CPR + SB		Sham + Vehicle	Sham + SB	CA/CPR + Vehicle	CA/CPR + SB		Sham + Vehicle	Sham + SB	CA/CPR + Vehicle	CA/CPR + SB
24 h	12	12	5	10	72 h	12	12		10	24 h	9	9	0	7	72 h	9	9		6
	12	12	8	8		12	12	5	7		9	9	4	3		9	9	1	2
	12	12	2	9		12	12		9		9	9	0	4		9	9		4
	12	12	8	7		12	12		6		9	9	2	4		9	9		2
	12	12	2	9		12	12		6		9	9	0	5		9	9		3
	12	12	3	8		12	12				9	9	1	1		9	9		
	12	12	3	6		12	12	2	5		9	9	0	5		9	9	0	3
	12	12	8	6		12	12	6			9	9	1	4		9	9	1	
	12	12		8		12	12		3		9	9		3		9	9		1
	12	12		8		12	12		7		9	9		1		9	9		1
	12	12		10		12	12		8		9	9		5		9	9		6
	12	12				12	12				9	9				9	9		
	12	12				12	12				9	9				9	9		
	12	12				12	12				9	9				9	9		
	12	12				12	12				9	9				9	9

### SB prevented microglial activation and regulated the microglial polarization after CA

**Fig. 3:**
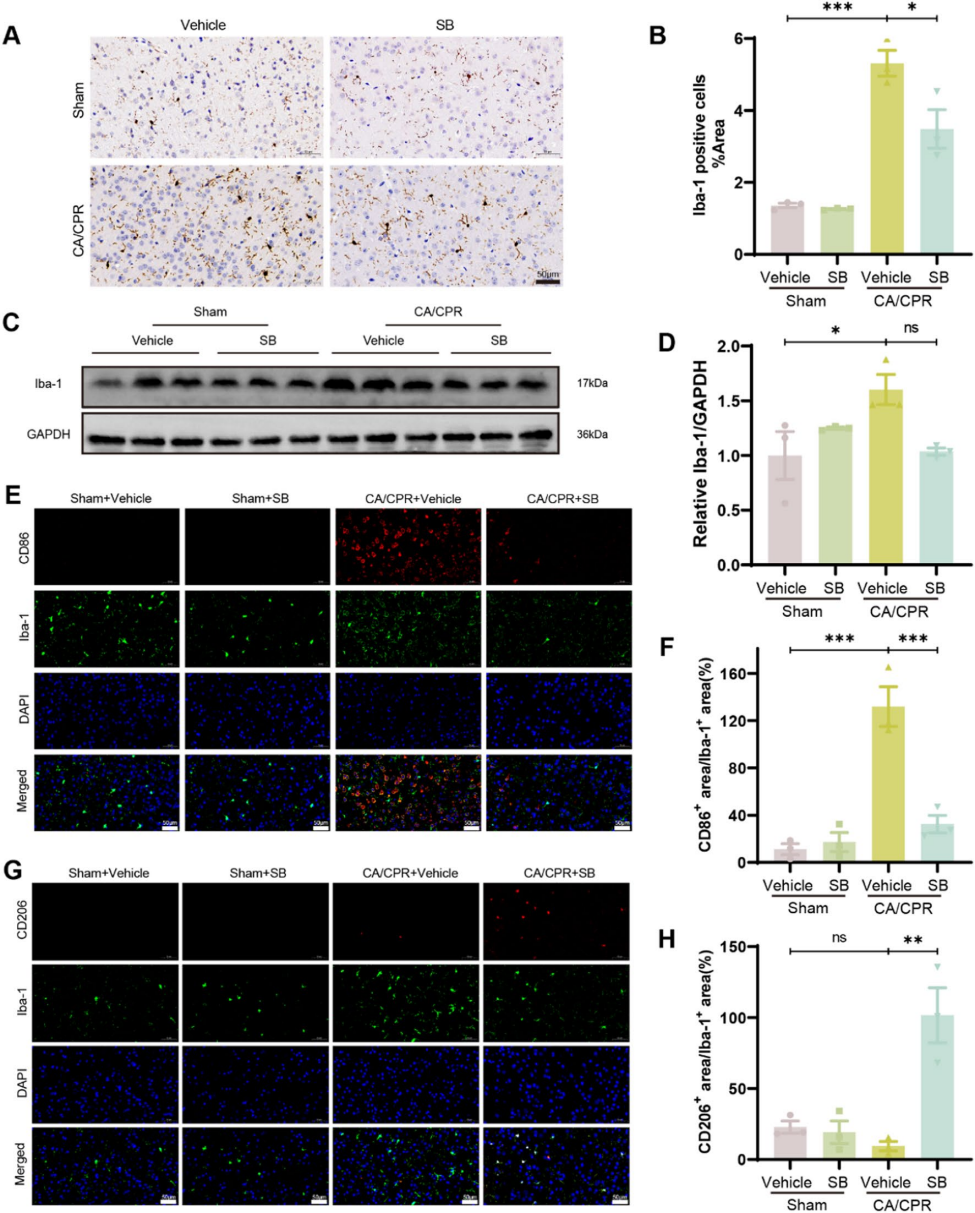
SB inhibited microglial activation and regulated the microglial polarization after CA. **A** Representative IHC staining images of Iba-1 in the cortex. **B** The statistical analysis of Iba-1-positive cell areas in the differently treated mice. **C** Representative western blotting patterns of Iba-1. **D** Iba-1 analysis in the cortex at 24 h post-CA/CPR. **E** Representative images of Iba-1 and CD86 double immunofluorescent staining in the cortex. **F** Quantification of CD86 cells/ microglial Iba-1 cells positive area. **G** Representative images of Iba-1 and CD206 double IF staining in the cortex. **H** Quantification of CD206 cells/microglial Iba-1 cells positive area. Scale bar: 50 µm (200 ×). Data are depicted as mean ± SEM (n = 3/group). ns, no significant, ***p < *0.001,* **p < *0.01,* *p < *0.05*

Microglia are essentially involved in neuroinflammatory response and their increased activation has been found to be associated with neuron loss following CA/CPR [10]. Therefore, this research study evaluated the impact of SB on microglial activation via IHC staining to observe the cortex levels of Iba-1 24 h post-CA/CPR (Fig. 3A). The sham group indicated very few Iba-1 positive microglia cells; however, the CA/CPR group had substantially increased expression of these cells (Sham + Vehicle vs CA/CPR + Vehicle, P = 0.0001), which were significantly reduced by SB therapy (CA/CPR + Vehicle vs CA/CPR + SB, P = 0.0167, Fig. 3B). To validate this data, WB analysis was carried out, which indicated that the level of Iba-1 expression was markedly reduced in the SB-treated group relative to the CA/CPR group 24 h post-CA (P = 0.061, Table 2, Fig. 3C–D). Furthermore, microglia polarization status was assessed using M1 (CD86) and M2 (CD206)-specific surface markers to elucidate whether SB promotes a protective role by affecting microglia polarization phenotypes. The proportion of CD86 and Iba-1 double-positive microglia area was substantially increased after CA/CPR (P = 0.0002) and decreased after SB treatment (P = 0.0006, Fig. 3E–H). Moreover, no significant alteration was found in the proportion of CD206 and Iba-1 double-positive microglia area in the CA/CPR and sham groups (P = 0.8172). However, SB treatment substantially enhanced the proportion of CD206 and Iba-1 double-positive microglia area (P = 0.0014), suggesting a phenotypic transition from M1 to M2 microglia. Overall, these data indicated that CA/CPR markedly induced microglial activation, while SB inhibited this activation and promoted microglia polarization toward the M2-type.

**Table 2:** The original grayscale values of Western Blot bands and the corresponding relative expression levels

TLR4-1	GAPDH-1	TLR4-2	GAPDH-2	TLR4-1/GAPDH-1	TLR4-2/GAPDH-2			Sham+Vehicle	Sham+SB	CA/CPR+Vehicle	CA/CPR+SB					
Animals																
8699.326	26790.33	9619.598	11497.36	0.324719	0.836679			0.324719	0.590954	1.117632	0.92305					
10811.74	23899.33	8827.719	10304.36	0.452387	0.856698			0.452387	0.663425	1.050995	0.804422					
14216.15	26619.62	5582.77	11344.6	0.534048	0.492108			0.534048	0.611695	1.205068	0.827484					
14480.03	24502.79	4703.062	11392.06	0.590954	0.412837			0.836679	0.412837	0.513492	0.699868					
15838.15	23873.33	3878.477	13264.65	0.663425	0.292392			0.856698	0.292392	0.922604	0.520771					
15791.03	25815.21	4199.648	13771.6	0.611695	0.30495			0.492108	0.30495	1.149012	0.608289					
27629.4	24721.38	7199.184	14020.06	1.117632	0.513492		mean	0.582773								
28684.33	27292.55	11924.01	12924.31	1.050995	0.922604		Relative TLR4/GAPDH	0.557196	1.014039	1.917782	1.583892					
28690.57	23808.26	13911.31	12107.18	1.205068	1.149012			0.776266	1.138393	1.803437	1.380334					
21882.28	23706.5	8049.719	11501.77	0.92305	0.699868			0.916391	1.049628	2.067817	1.419908					
19676.98	24461.03	6267.426	12034.89	0.804422	0.520771			1.435686	0.7084	0.881117	1.200927					
19356.62	23392.13	8138.77	13379.77	0.827484	0.608289			1.470037	0.501725	1.583127	0.893609					
								0.844425	0.523274	1.971629	1.043784					

TLR4-1	GAPDH-1	TLR4-2	GAPDH-2	TLR4-3	GAPDH-3	TLR4-1/GAPDH-1	TLR4-2/GAPDH-2	TLR4-3/GAPDH-3	mean				Control	Control+SB	OGD/R	OGD/R + SB
Cells																
27976.41	33896.85	31099.95	35836.87	19946.22	32070.29	0.825339	0.86782	0.621953	0.771704	1.069502	1.12455	0.805948	1.069502	1.102998	1.493291	1.059064
29697.41	34889.36	29027.66	33881.51	29301	35546.65	0.851188	0.85674	0.824297		1.102998	1.110193	1.068152	1.12455	1.110193	1.547392	1.010152
41079.39	35647.46	40391.95	33825.46	44353.84	34496.8	1.152379	1.194129	1.285738		1.493291	1.547392	1.666102	0.805948	1.068152	1.666102	1.370118
33279.17	40719.22	24264.34	31126.54	36128.58	34169.77	0.817284	0.779539	1.057326		1.059064	1.010152	1.370118

### SB inhibited neuroinflammation via the TLR4/MyD88/NF-κB pathway after CA

**Fig. 4:**
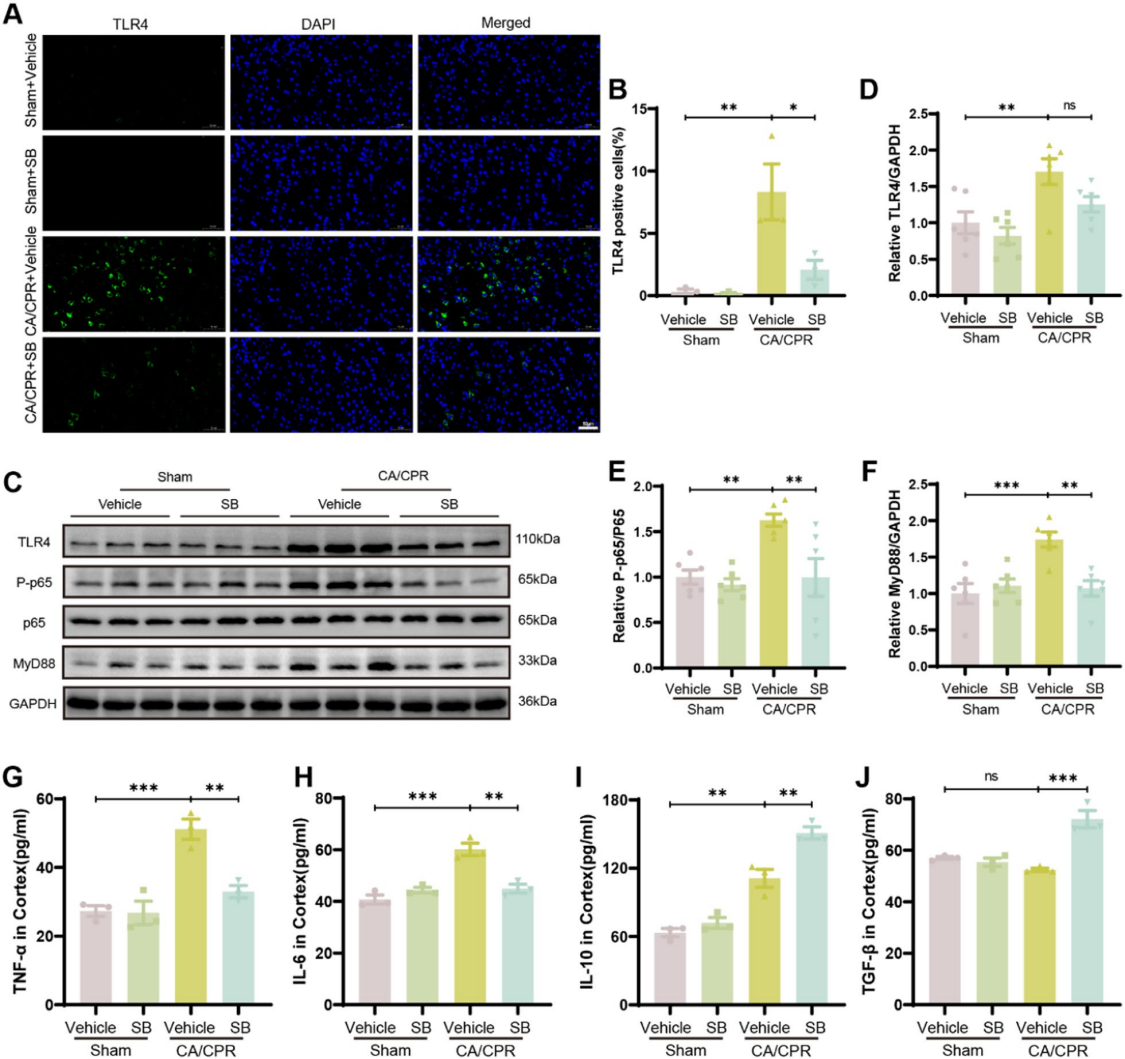
SB reduced the neuroinflammatory response by suppressing the signaling pathway of TLR4/MyD88/NF-κB. **A** Representative IF staining images of toll-like receptor 4 (TLR4) in the cortex. **B** Quantification of the number of TLR4 positive cells of diverse groups. **C**–**F** Representative images of blots and quantitative assessment of MyD88, TLR4, and P-NF-κB protein expression in different groups. **G**–**J** The levels of TGF-β, IL-6, TNF-α, and IL-10 in the cortex were identified by ELISA assay. Scale bar: 50 µm (200 ×). Data are displayed as mean ± SEM (n = 3/group). ns, no significant, ****p < *0.0001,* ***p < *0.001*, **p < *0.01*, *p < *0.05*

Previous research has revealed that after ischemic stroke, activated the signaling pathway of TLR4/MyD88/NF-κB increases the production of inflammatory mediators [35]. A recent study demonstrated that in Parkinson’s disease, SB improves brain function by inhibiting microglia-mediated neuroinflammation by TLR4/MyD88/NF-κB signaling pathway [36]. Thus, it was hypothesized that SB can prevent CA/CPR-induced microglial inflammation by modulating the TLR4/MyD88/NF-κB signaling pathway. To test this assumption, the levels of P-p65 (P-NF-κB), MyD88, TLR4, and p65 (NF-κB) proteins in the cortical tissue of mice 1-day post-CA/CPR were assessed via WB and IF staining. The IF data revealed that TLR4^+^ cells were elevated in the CA/CPR mice compared to the sham group (P = 0.0065). In contrast, treatment with SB reduced the number of TLR4^+^ cells (CA/CPR + Vehicle vs CA/CPR + SB, P = 0.0253, Fig. 4A, B). Moreover, WB analysis revealed that CA/CPR markedly enhanced TLR4, P-p65/p65, and MyD88 protein levels relative to the sham group (P = 0.01, P = 0.0078, P = 0.0007, respectively), whereas the overall levels of NF-κB protein remained constant (P = 0.9997, Table 2, Fig. S1A). Similarly, SB administration substantially alleviated the expression levels of these proteins (P = 0.1389, P = 0.0074, P = 0.002, respectively, Table 2, Fig. 4C–F). To elucidate the levels of inflammation in mouse brains, the expressions of TGF-β and IL-10 (anti-inflammatory cytokines) and TNF-α and IL-6 (pro-inflammatory cytokines) at 24 h post-CA/CPR were detected in brain homogenates. The ELISA revealed that relative to the sham group, administration of SB reduced the CA/CPR-induced high TNF-α(CA/CPR + Vehicle vs CA/CPR + SB, P = 0.0042) and IL-6(CA/CPR + Vehicle vs CA/CPR + SB, P = 0.0014) expressions and elevated IL-10(CA/CPR + Vehicle vs CA/CPR + SB, P = 0.0045) and TGF-β(CA/CPR + Vehicle vs CA/CPR + SB, P = 0.0007) levels in the cortex (Fig. 4G–J). Altogether, these findings suggest that the signaling pathway of TLR4/MyD88/NF-κB may be associated with the neuroinflammatory response generated by CA.

### SB alleviated CA-induced intestinal damage and systemic inflammatory response

**Fig. 5:**
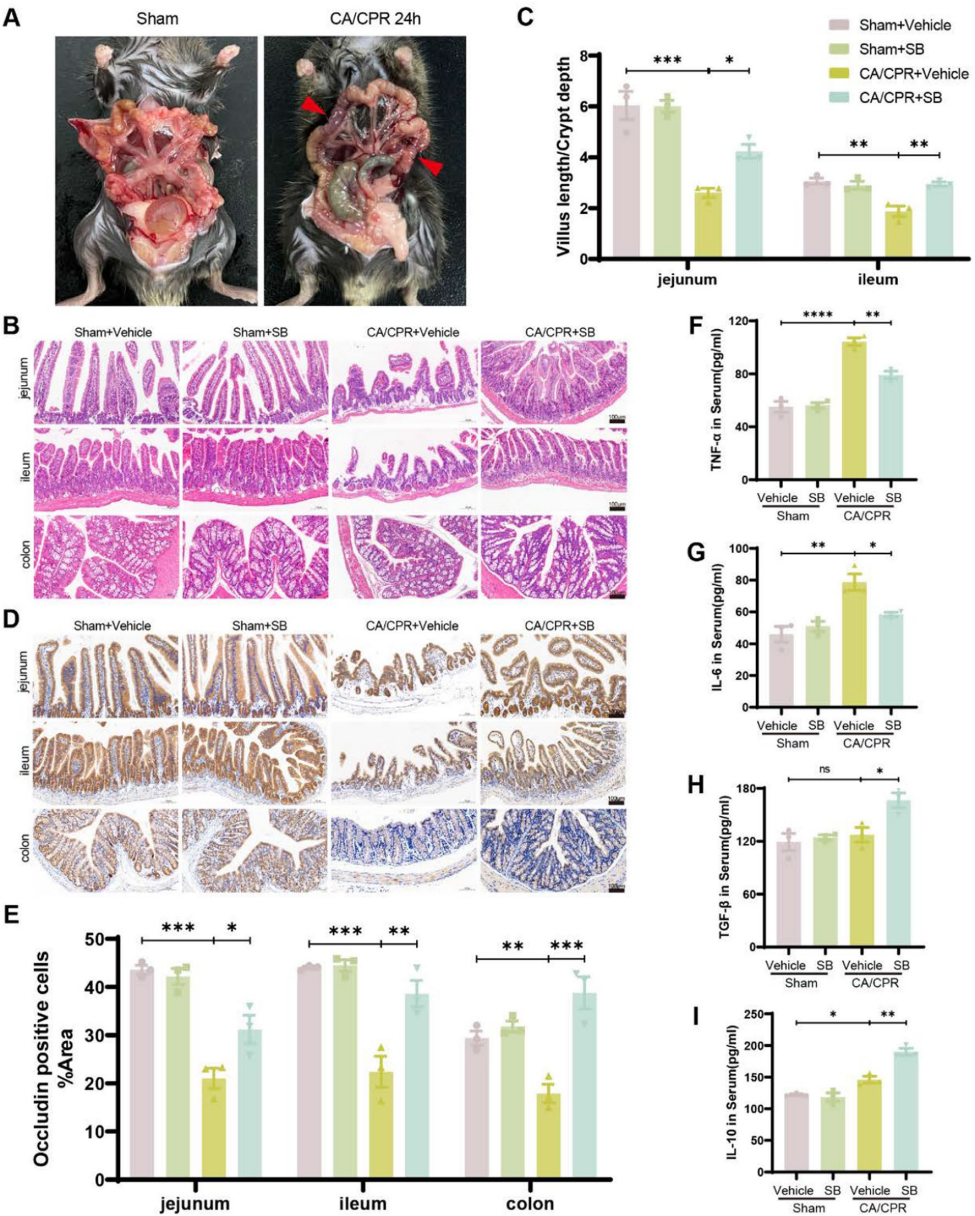
SB attenuated CA/CPR-induced gut injury and systemic inflammation. **A** Appearance of intestinal tissue in sham and CA/CPR groups. Red arrows = the ischemic lesion area. **B** Representative H&E stained images of Jejunum, ileum, and colon tissue morphology. **C** Quantification of the ratio of jejunum and ileum villus length and crypt depth. **D** Representative IHC staining of occludin in the intestine. **E** The statistical analysis of the proportion of the area occupied by positive occludin cells in differently treated mice. **F-I** Serum levels of TGF-β, IL-6, TNF-α, and IL-10 were measured by ELISA assay. Scale bar: 100 µm (100 ×). Data were depicted as mean ± SEM (n = 3/group) ns, no significant, ****p < *0.0001,* ***p < *0.001*, **p < *0.01*, *p < *0.05*

Intestinal I/R injury can impair intestinal mucosal epithelial barrier function and endotoxin translocation, thereby exacerbating distal organ harm [3]. Here, after mice dissection (Fig. 5A and Fig. S1B), the intestinal tubes were collected. The data revealed that the sham group had neat positioned peritoneal cavity without swelling and dilatation. At 30 min post-ROSC, the intestinal walls of the mice indicated congestion and inflammation. Furthermore, at 24 h post-CA, several intestinal segments were observed to be swelled and dilated, indicating noticeable bruising. Moreover, at 72 h post-ROSC, the intestinal tubes in the peritoneal cavity were disorganized, blackened, and thinned with wide-scale necrosis.

The intestinal tissue condition was examined via H&E staining and the sham mice had normal structure, characterized by orderly distributed villi, tightly connected and intact epithelium, and no inflammatory cell infiltration. Whereas, CA/CPR mice indicated irregular and shortened villi, partial exfoliation of submucosa and muscular layers, markedly increased mucosal damage, and infiltration of inflammatory cells. However, SB-treated mice showed alleviation of these pathological changes (Fig. 5B). Further, statistical analysis of the intestinal villus height-to-depth ratio (Fig. 5C) indicated that the CA/CPR group had a lower ratio than the sham group (jejunum P = 0.0005, ileum P = 0.0022); however, it was slightly improved post-SB treatment (jejunum P = 0.0384, ileum P = 0.0042) but less than the sham group. Moreover, the IHC analysis was carried out to detect occludin expression, which is necessary for barrier function and integrity. The CA/CPR group had shallower positive cell staining than the sham group (Fig. 5D), which became deep after SB treatment. Moreover, statistical analysis revealed that SB significantly increased the area of occludin-positive cells (CA/CPR + Vehicle vs CA/CPR + SB, jejunum P = 0.0141, ileum P = 0.0064, colon P = 0.0002, Fig. 5E). The disruption of the intestinal barrier may promote a systemic inflammatory response in the CA/CPR model [37]. Therefore, the serum levels of inflammatory cytokines were evaluated by ELISA, which revealed elevated TNF-α (P < 0.0001), IL-6(P = 0.0017), and IL-10(P = 0.047) levels, with no significant change in TGF-β (P = 0.8759) levels between the sham and CA/CPR groups. However, SB administration decreased TNF-α (P = 0.0018) and IL-6(P = 0.0274) levels while increasing TGF-β (P = 0.0299) and IL-10(P = 0.0014) levels (Fig. 5F–I). Overall, these results indicated that SB treatment alleviates CA-induced intestinal injury and enhances intestinal permeability, thus inhibiting systemic inflammation.

### SB altered the intestinal microbiota composition after CA

**Fig. 6:**
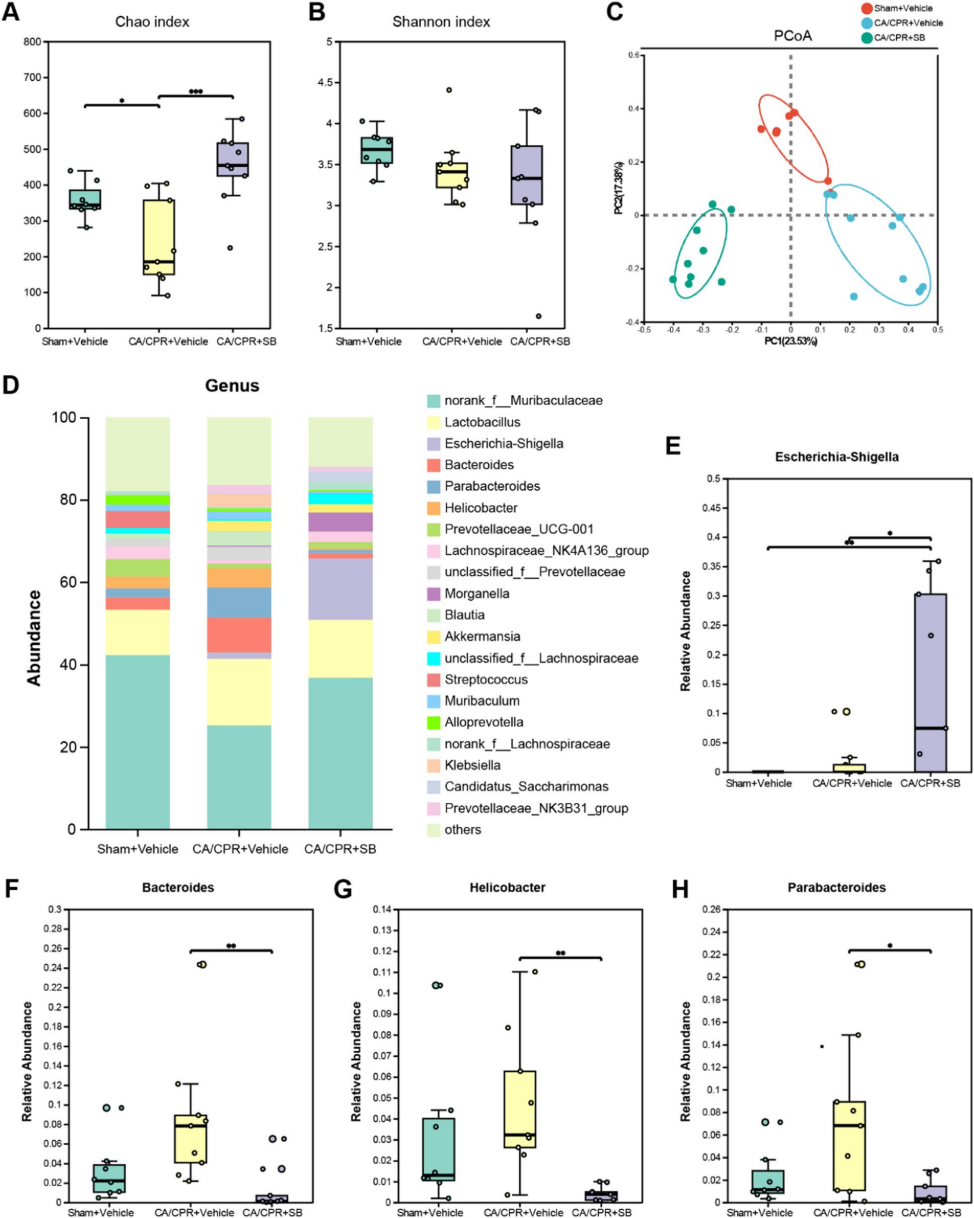
SB modulated the composition of gut microbiota after CA/CPR. **A-B** Alpha diversity is presented by the Chao and Shannon index. **C** Beta diversity presented by PCoA; **D** Community structure component diagram. **F-G** The abundance of *Bacteroides*, *Helicobacters, Escherichia-Shigella*, and *Parabacteroides* in Sham + Vehicle, CA/CPR + Vehicle, CA/CPR + SB groups. Data were displayed as mean ± SEM (n = 8—9/group) ***p < *0.001,* **p < *0.01*, *p < *0.05*

Dysbiosis of intestinal flora is markedly linked with the prognosis of cerebral ischemia injury [38]. Therefore, this research study carried out 16S rDNA sequencing of fecal samples to analyze the changes in microbiota composition. The data revealed a substantial difference in community richness (Chao index) (P = 0.0054); however, there was no significant difference in community diversity (Shannon index) (P = 0.2062, Fig. 6A, B). PCoA and NMDS analyses revealed distinct differences in the community of gut microbiota between the 3 groups (P = 0.001, Fig. 6C, Fig. S2A). The Venn diagram indicates the three diverse groups of shared and distinct operational taxonomic units (OTUs), with all groups collectively sharing 335 OTUs. Furthermore, the sham and SB groups had 114 common OTUs, whereas sham and CA only indicated 43 shared OTUs. (Fig. S2B). The differences in the top 10 species with average abundance at the phylum level were detected between the groups. The results indicated that CA elevated the relative abundance of Campilobacterota, Verrucomicrobiota, Deferribacterota, Patescibacteria, and Proteobacteria, compared with the sham group. Whereas SB further markedly enhanced the abundance of Proteobacteria and Patescibacteria but decreased Campilobacterota (p < 0.01 for each, Fig. S2C). At the genus level, the top 20 most abundant bacterial genera were identified and *norank_f__Muribaculaceae* and *Lactobacillus* were the dominating genera in all the three groups (Fig. 6D). After CA/CPR, the abundance of *Prevotellaceae_UCG-001* was markedly down-regulated (p < 0.01), while that of *Bacteroides, Helicobacters, Parabacteroides, Klebsiella,* and *Prevotellaceae*_*NK3B31_group* were upregulated; however, they were not statistically significant. Moreover, SB treatment substantially decreased the relative abundance of pathogens, such as *Bacteroides* (p < 0.01)*, Helicobacters* (p < 0.01)*,* and *Klebsiella* (p < 0.05). Furthermore, SB intervention upregulated *Escherichia-Shigella* (p < 0.05, Fig. 6E–H, Fig. S2D-F). Taken together, these observations demonstrated that SB treatment can modify microbiota composition and increase gut flora richness in KCl-induced CA mice.

### SB altered the content of SCFA after CA

**Fig. 7:**
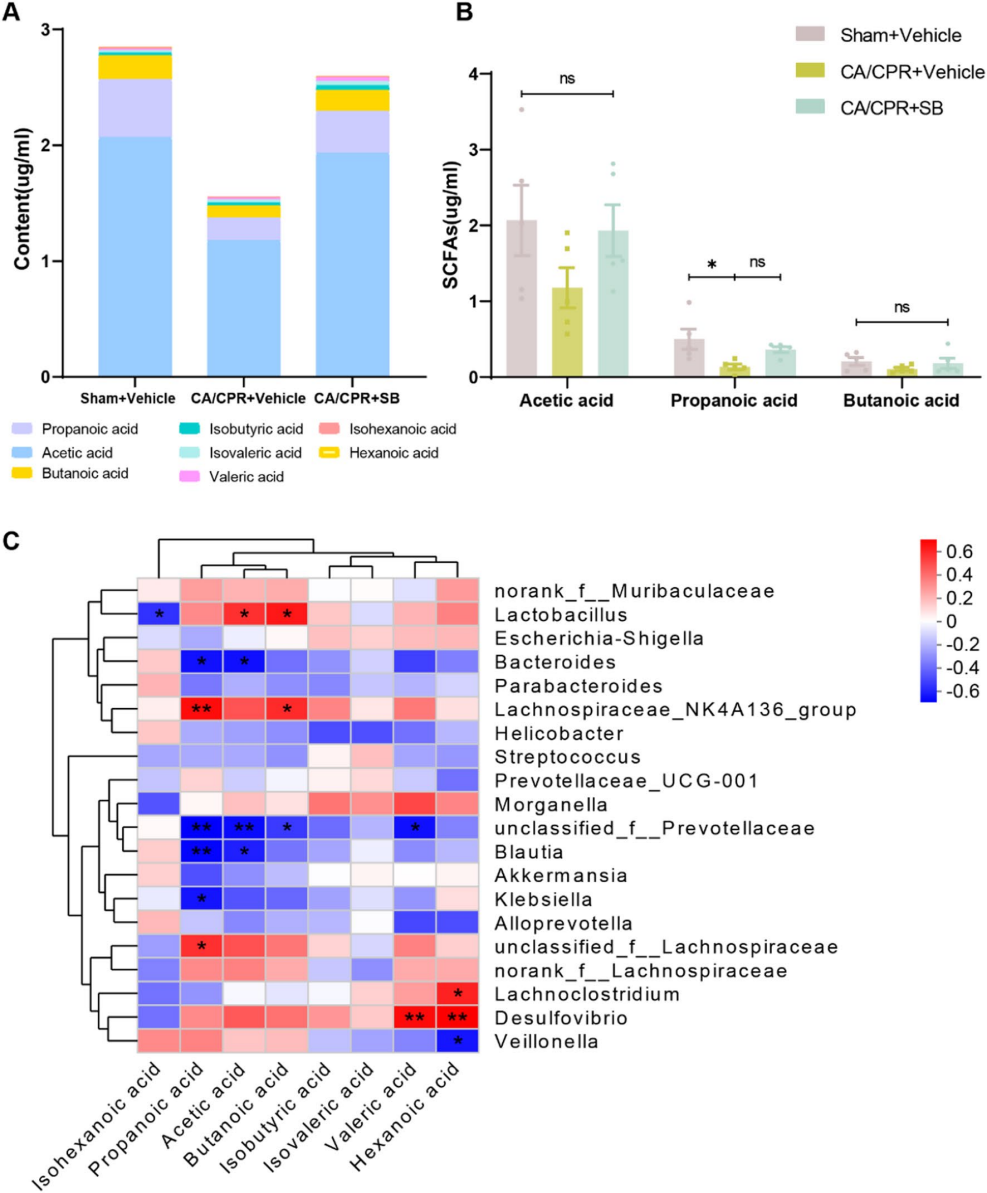
SB changed SCFA production after CA/CPR. **A** The levels of fecal SCFA in Sham + Vehicle, CA/CPR + Vehicle, CA/CPR + SB groups. **B** Fecal levels of acetic, propanoic, and butanoic acid. **C** The correlation heatmap between the fecal SCFA levels and the top 20 most abundant genera is based on the Spearman correlation network data. Blue, negative correlation; Red, positive correlation. Data are displayed as mean ± SEM (n = 5/group). ns, no significance, **p < *0.01,* *p < *0.05*

Total fecal SCFA concentrations were markedly reduced in the CA mice than in the sham mice, whereas SB increased the total SCFA levels (Fig. 7A). Furthermore, the CA group had reduced fecal levels of acetic (P = 0.2377) and butanoic acids (P = 0.3866) but only propanoic acid (P = 0.0215) concentrations were markedly reduced. Moreover, an increasing trend was observed in acetic (P = 0.3447), butanoic (P = 0.57), and propanoic acid (P = 0.1643) levels without significance between the CA/CPR and CA/CPR + SB groups (Fig. 7B). The correlation heatmap analysis revealed the fecal levels of propanoic and butanoic acids were substantially and positively associated with the abundance of *Lachnospiraceae_NK4A136_group* (P = 0.007, P = 0.0234, respectively), while negatively linked with *unclassified_f__Prevotellaceae* (P = 0.0054, P = 0.0462, respectively). Similarly, propanoic acid indicated statistically significant negative associations with *Blautia, Klebsiella,* and *Bacteroides* (P = 0.0064, P = 0.0129, P = 0.0115, respectively). Whereas acetic acid had a positive relationship with *Lactobacillus* abundance (P = 0.0272). Moreover, a negative correlation was observed between acetic acid and *unclassified_f__Prevotellaceae, Blautia,* and *Bacteroides* (P = 0.0087, P = 0.0196, P = 0.0109, respectively), along with a nonsignificant correlation between acetic acid and *Klebsiella* (P = 0.0913, Fig. 7C).

### SB promoted M1 to M2 microglial phenotypic polarization in vitro, thus reducing inflammation

**Fig. 8:**
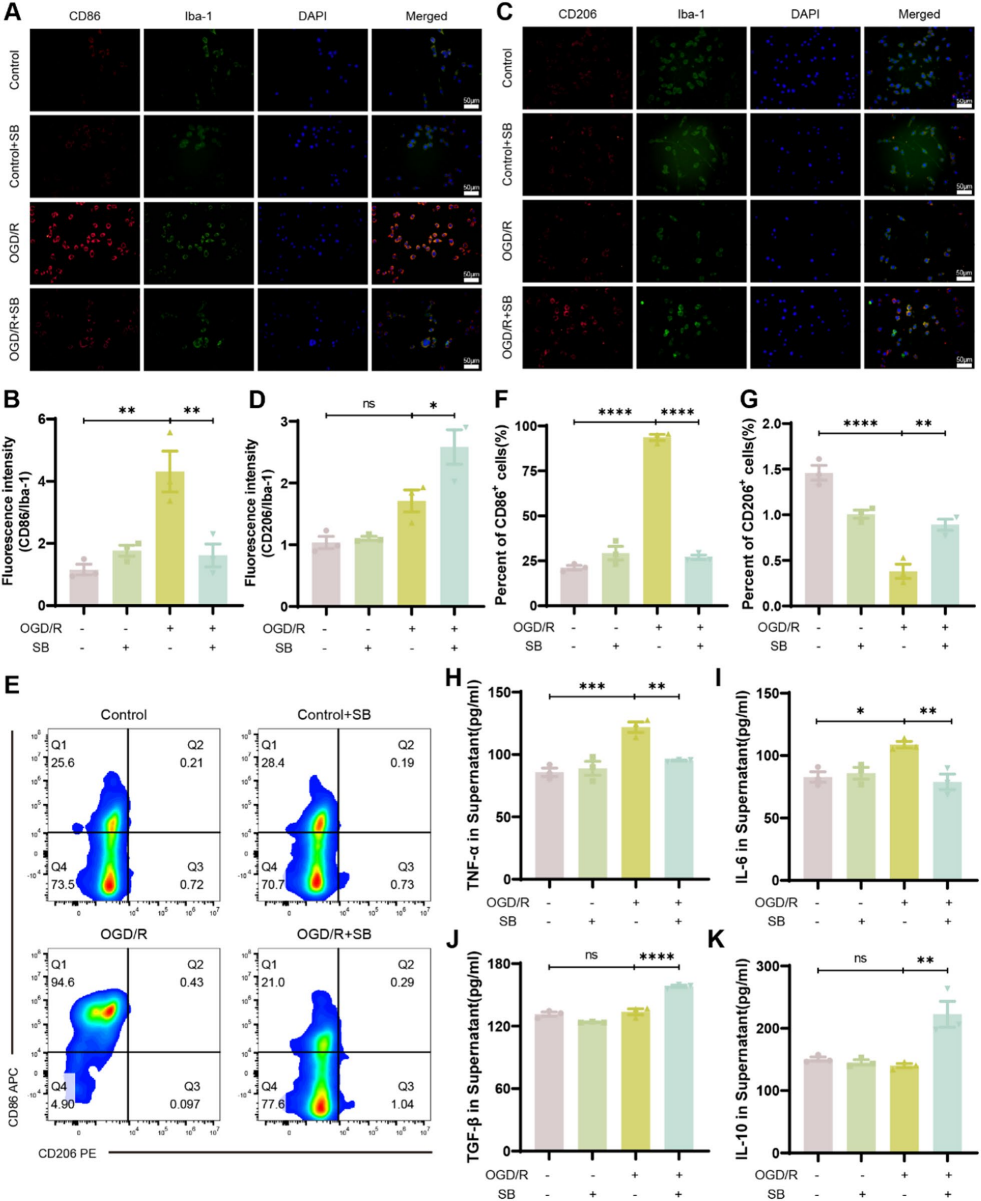
SB mediated the activation of microglia M1/M2 polarization and attenuated microglial inflammation. **A** Representative images of CD86 and Iba-1 double immunostaining. **B** Quantitative analysis of fluorescence intensity in control or OGD/R BV2 cells with or without SB treatment. **C** Representative images of CD86 and Iba-1 double immunostaining and **D** quantitative analysis of fluorescence intensity in control or OGD/R BV2 cells; treated or not-treated with SB. **E–G** The percentage of CD86 and CD206 positive BV2 cells detected by PE/APC staining with flow cytometric analysis. **H-I** TNF-α, IL-6, TGF-β, and IL-10 levels in supernatants were assessed by ELISA. Scale bar: 50 µm (200 ×). Data were displayed as mean ± SEM (n = 3/group). ns, no significant, ****p < *0.0001,* ***p < *0.001,* **p < *0.01*, *p < *0.05*

The anti-inflammatory properties of SB on microglia were further evaluated in vitro using mouse microglial BV2 cells. The CCK8 assay revealed that OGD/R markedly reduced BV2 cell viability. Furthermore, SB did not affect BV2 cell viability at concentrations below 250 μM. These data suggest that OGD/R causes cytotoxicity, whereas SB has no cytotoxic effects on BV2 cells at concentrations between 0 μM and 250 μM (Fig. S3A, B). Moreover, SB increased BV2 cell viability induced by OGD (2 h)/R (24 h). Therefore, 10 μM SB was selected as the effective treatment dose for subsequent experiments (Fig. S3C). The IF analysis indicated markedly increased fluorescence density of CD86(P = 0.0021) and CD206(P = 0.0965) following OGD/R induction (Fig. 8A and C). It was observed that SB treatment increased the fluorescence density of CD206 (P = 0.0306) while decreasing the CD86 fluorescence density (P = 0.0057, Fig. 8B and D). Flow cytometry further confirmed that OGD/R promoted CD86 and suppressed CD206 expression of BV2 cells (P < 0.0001 for each), while SB treatment significantly suppressed CD86 (P < 0.0001) and slightly promoted CD206 levels (P = 0.0032, Fig. 8E–G). Furthermore, BV-2 cells indicated enhanced levels of TNF-α (P = 0.0008) and IL-6(P = 0.0169) after OGD/R; however, SB reduced these levels (OGD/R vs OGD/R + SB, TNF-α P = 0.0055, IL-6 P = 0.0077), while also enhancing the levels of TGF-β(P =  < 0.0001) and IL-10(P = 0.0029). Overall, these data demonstrated that SB promotes microglial polarization from the M1 to M2 phenotype, thereby reducing inflammation in vitro.

### 3.8. SB protected against microglial-mediated neurotoxicity by suppressing NF-κB p65 protein nuclear translocation and the TLR4/MyD88/NF-κB signaling pathway in BV2 microglial cells

**Fig. 9:**
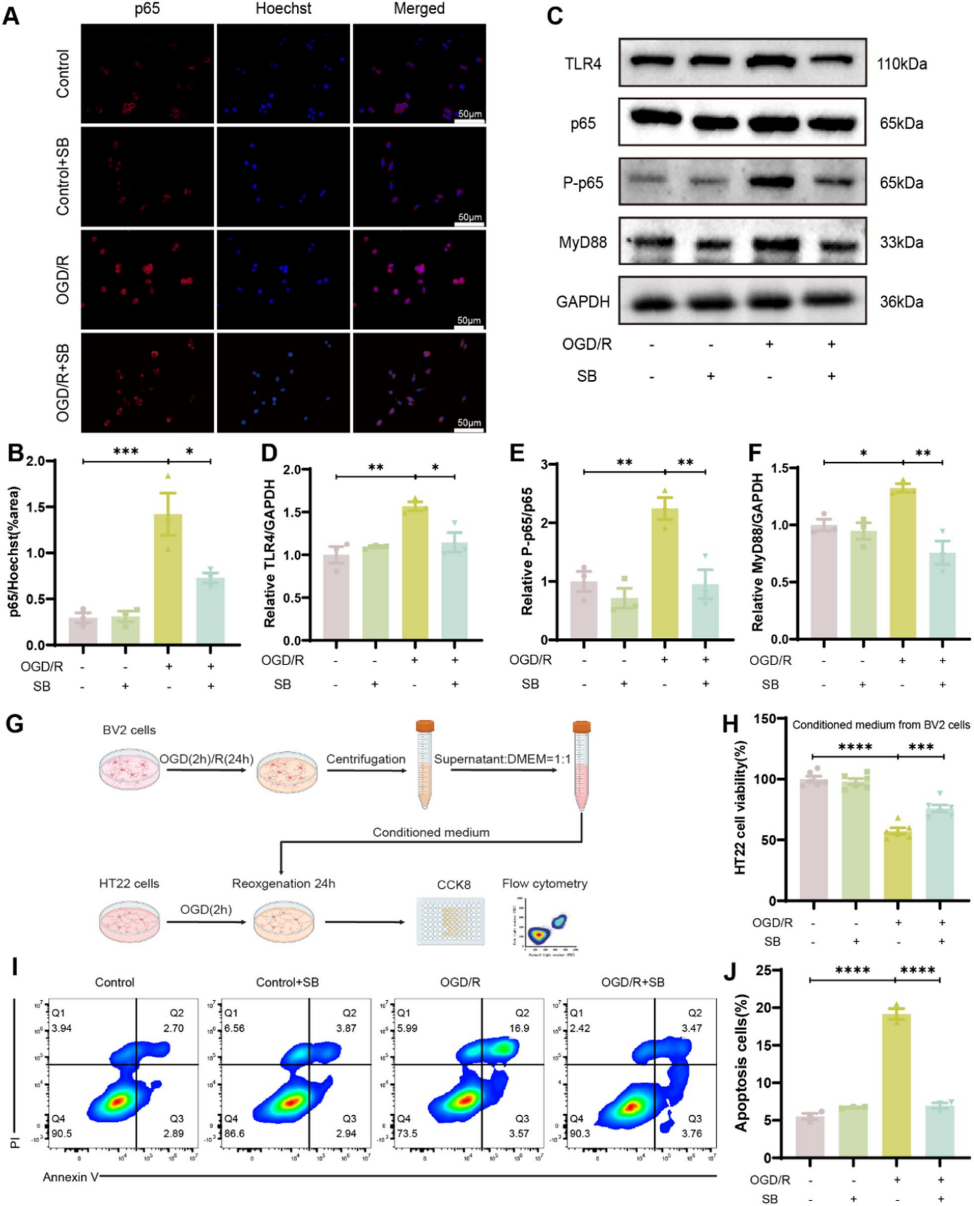
SB had neuroprotective effects by suppressing the TLR4/MyD88/NF-κB signaling pathway. **A** Representative images and **B** quantitative analysis of the area occupied by NF-κB p65-positive cells in the nuclei accessed by IF staining in control or OGD/R BV2 cells; treated or not-treated with SB. **C** Representative blot images of **D** TLR4, **E** P-p65/p65, and **F** MyD88. **G** Schematic diagram of the co-culture system. **H** Cell viability accessed by CCK8 assay (n = 6/group). **I, J** The proportion of apoptosis in the indicated group of HT22 cells was analyzed by flow cytometry. Scale bar: 50 µm (200 ×). Data are displayed as mean ± SEM (n = 3/group). ****p < *0.0001,* ***p < *0.001,* **p < *0.01*, *p < *0.05*

To identify the mechanism of SB on microglia inflammatory response to OGD/R, IF staining analysis was carried out to explore the nuclear translocation of NF-κB p65 protein in BV2 cells from different intervention groups (Fig. 9A). The data revealed that SB markedly blocked OGD/R-induced p65 nuclear translocation in BV2 cells (OGD/R vs OGD/R + SB, P = 0.0181, Fig. 9B). Furthermore, whether SB modulates the TLR4/MyD88/NF-κB pathway in OGD/R-stimulated BV2 cells was also assessed. The results indicated markedly enhanced TLR4, P-p65/p65, and MyD88 expression in the OGD/R group than the control group (P = 0.0042, P = 0.0087, P = 0.0447, respectively); however, SB treatment reduced these proteins (P = 0.0228, P = 0.007, P = 0.002, respectively, Table 2, Fig. 7C–F). To investigate the neuroprotective effect of SB, OGD/R-stimulated HT22 cells were treated with conditioned media obtained from BV2 cells for 24 h. Then, cell viability and apoptosis were assessed (Fig. 9G), which showed that conditioned media from OGD/R-induced BV2 cells reduced the viability of HT22 cells (Control vs OGD/R, P < 0.0001), which was mitigated by SB treatment (OGD/R vs OGD/R + SB, P = 0.0003, Fig. 9H). Moreover, SB reduced OGD/R-induced neuronal apoptosis (OGD/R vs OGD/R + SB, P < 0.0001, Fig. 9I-J). Altogether, these findings indicated that SB’s neuroprotective effect is mediated by inhibiting p65 nuclear translocation and the TLR4/MyD88/NF-κB signaling pathway stimulation, which regulate microglia phenotypic conversion and inflammatory responses.

## Discussion

**Fig. 10:**
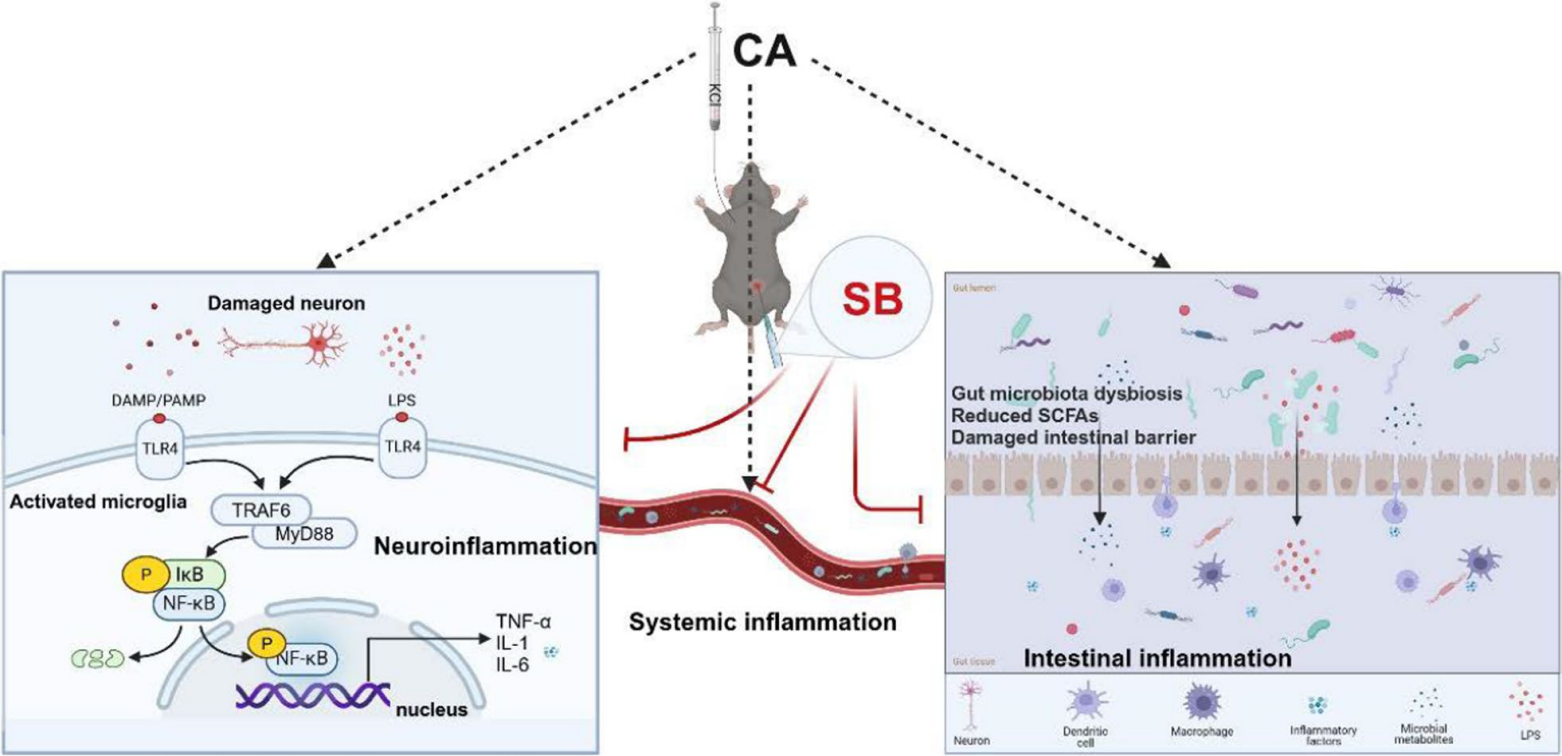
SB protects against CA/CPR via the microbiome-gut-brain axis

This study indicated that SB can effectively protect against CA/CPR by inhibiting bacteria dysbiosis, elevating SCFA levels, alleviating intestinal barrier impairment, reducing systemic and neuroinflammatory responses, and promoting an M1 to M2 microglial phenotypic transformation, thus protecting the brain against global ischemic stimulation. Furthermore, it was found that SB may promote anti-inflammatory effects by downregulating NF-κB phosphorylation in the nucleus and the signaling pathway of TLR4/MyD88/NF-κB (Fig. 10).

Research suggests that the most effective and essential CA treatment is high-quality and timely CPR, which restores the blood supply; however, recanalization of ischemic cerebral vessels stimulates various mechanisms contributing to secondary brain injury. The reperfusion brain injury pathogenesis includes microglial activation, which releases pro-inflammatory chemokines and cytokines [39]. Furthermore, microglia identify and modulate neuronal activity, an excessive inflammatory response by microglia can result in neuronal death [40]. Previous studies were primarily focused on SB’s anti-inflammatory effects [41] and how it promoted the M1 pro-inflammatory to M2 anti-inflammatory microglial type [42] in the in vivo and in vitro regional cerebral I/R models [43]. Here, it was hypothesized that SB can protect neurons from neuroinflammatory damage after CA/CPR-induced global I/R injury. The results verified this hypothesis, indicating microglial activation after CA/CPR, which was suppressed after SB treatment. Furthermore, SB also altered the microglial polarization state to reduce neuroinflammation, reduced neuronal death and apoptosis in vivo*,* and protected HT22 cells from the neurotoxic conditioned media in vitro. Overall, these data indicated that SB alleviates brain damage post-CA by inhibiting microglia inflammation. However, it is controversial to categorize microglia into only M1 or M2 phenotypes and single-cell sequencing should be employed for assessing more detailed subtypes and precise microglial mechanisms in the future.

Toll-like receptor 4 (TLR4) is mostly expressed on microglial surfaces, whereas NF-κB is a downstream effector within the TLR4 signaling cascade, controlled by positive feedback mechanisms [44]. TLR4 interacts with damage-associated molecular patterns (DAMP) or pathogen-associated molecular patterns (PAMP) to activate and promote the nuclear translocation of NF-κB via MyD88 binding, thereby stimulating inflammatory response by influencing the levels and release of inflammatory cytokines and mediators [45]. Several studies have indicated that after CA, the activated TLR4 signaling pathway stimulates the development and acceleration of CNS inflammation [46]. Here, the data revealed elevated expression of TLR4 pathway-related; however, SB inhibited this increase in vivo and in vitro. Moreover, there was substantial nuclear translocation of NF-κB in OGD/R-induced BV2 cells, which was also reduced by SB. Therefore, it was inferred that the protective effect of SB is markedly linked with the TLR4/MyD88/NF-κB pathway.

Commensal gut bacteria has been found to affect the host immune system and disease processes in multiple organs, including the brain. Recently, a study indicated that asphyxia-induced CA rats had diminished alpha diversity and altered GM composition relative to the sham group [47]. However, whether the microbiome influences the prognosis of acute brain injury remains elusive [48]. In this investigation, 16S rDNA sequencing was carried out on mice feces 24 h post-CA, which revealed significant alterations in the intestinal bacterial composition of the CA group to that of the sham group. At the genus level, *Bacteroides, Parabacteroides, Helicobacter, Blautia, Akkermansia*, and *Muribaculum* were significantly increased, while *Prevotellaceae_UCG-001* and *Lachnospiraceae_NK4A136_group* were reduced in the CA mice relative to the sham mice. Furthermore, SB treatment reversed these alterations. However, the abundance of *Escherichia-Shigella* and *Morganella*, regarded as pathogens and opportunistic microorganisms [49], was further enhanced after SB intervention. SCFA are important metabolites produced by the gut microbiome, mostly comprising acetic acid, butyric acid, and propionic acid, which modulate inflammation and affect the intestinal epithelial barrier [50]. It has been observed that the fecal levels of SCFA reduce substantially after stroke, and the severity of stroke was positively correlated with the reduction of SCFA [27]. Therefore, the transplantation of SCFA-rich fecal microbiota and butyric acid supplements can effectively improve the neurological recovery from ischemic stroke [30]. Here, it was found that the CA group had reduced levels of total SCFA, especially propionic acids, whereas SB therapy increased acetic, propionic, and butyric levels. Therefore, modulating GM to recover the SCFA levels may be an effective therapeutic approach for addressing PCABI. Currently, studies in the field of CA and on the specific regulatory mechanisms of intestinal flora’s protective effect on brain injury after CA/CPR are scarce and warrant further research.

Following CA/CPR-induced systemic I/R, PCAS results in multiple orange injuries, where the intestine injury is considered a critical component exacerbating the poor prognosis of these patients. After CPR onset, intestinal blood flow decreases to < 5% of the cardiac output, which markedly reduces intestine protection compared to the brain during CA/CPR [37]. A study indicated that among 214 PCAS patients who were admitted to the ICU, 56.5% suffered from ischemic damage to the upper gastrointestinal tract, with severe ischemic lesions strongly correlated with death [24]. Aberrant levels of gastrointestinal function biomarkers, such as intestinal fatty-acid binding protein (IFABP) and diamine oxidase (DAO) can indicate gastrointestinal tract injury following CA [51, 52]. A study on the porcine CA model revealed that 24 h post-resuscitation, there was significantly enhanced apoptosis in ileum tissues [53]. These data were consistent with the current study, which showed severe intestinal injury and markedly elevated intestinal permeability after CA/CPR, which was characterized by pronounced inflammatory edema, disrupted mucosal integrity, and reduced occludin expression. Furthermore, SB treatment alleviated these pathological alterations. Since CA and resuscitation promote a systemic inflammatory response during the acute phase [11], the serum expression of inflammatory factors in CA mice was assessed. The data revealed that pro-inflammatory cytokines levels were elevated and SB invention reduced these levels while increasing the expression of anti-inflammatory factors. Overall, these results suggested that SB may inhibit systemic inflammatory responses by protecting the intestine.

This research study has certain limitations. (1) Only immortalized cell lines (BV-2 and HT22) rather than primary cells from neonatal mice were employed in this study. In the future, we plan to separate primary microglial and neuronal cells for further studies. (2) This study only analyzed the anti-inflammation effects of SB on microglial for a short period (24 h). Therefore, the long-term effects of SB on the microglial polarization state through the TLR4/MyD88/NF-κB signaling pathway remain uncertain. (3) Although the alterations in GM composition across each group were discussed, the involvement of GM was not validated. (4) SB inhibition of microglial activation was found to promote a protective effect on neurons; however, the impact of alterations in neuronal signaling on microglial activation was not determined. There is a lot of evidence supporting the mutual interactions between neurons and microglia in maintaining CNS homeostasis; however, the immune regulatory functions of neurons are largely under-toned [40]. Therefore, further research is warranted to clarify the impact of injured neurons on microglia following CA. (5) SB exerts neuroprotective effects through multiple pathways, including epigenetic regulation of microglial phenotypes [33], modulation of immune homeostasis [54], and regulation of gut microbiota [30]. At present, we cannot definitively conclude that SB acts primarily on microglia to trigger a cascade of systemic and gut microbiota changes, or whether the effects begin in the peripheral nervous system or gut microbiota and subsequently affect microglia. Nor can we confirm if these are independent effects. More rigorous experiments need to be designed in the future to elucidate the specific mechanisms of protective action of SB.

In summary, this study indicated that SB alleviated the CA/CPR-induced GM dysbiosis, perturbed SCFA levels, intestinal mucosal damage, increased levels of inflammatory cytokine, and neuronal injury in mice. Furthermore, the anti-inflammatory mechanism of SB might be related to the modulation of microglia polarization balance, which is attributed to the inhibition of the TLR4/MyD88/NF-κB signaling pathway and NF-κB nuclear translocation. To the best of our knowledge, this study is the first to indicate that SB protects against CA/CPR-induced neuronal injury, possibly by normalizing GM dysbiosis. Future studies should assess the detailed underlying mechanisms.

## Supplementary Information

Additional file 1.

## Data Availability

All methods and materials used are described in the manuscript and data and analyses can be obtained from the corresponding or first author upon reasonable request.
