# Supplementary material for: Sodium butyrate attenuates microglia-mediated neuroinflammation by modulating the TLR4/MyD88/NF-κB pathway and microbiome-gut-brain axis in cardiac arrest mice
Source: Mol Brain. 2025 Feb 17;18:13. doi: 10.1186/s13041-025-01179-w (PMC11834616; doi:10.1186/s13041-025-01179-w)
Supplement: Supplementary file 1 — Additional file 1. [file 13041_2025_1179_MOESM1_ESM.pdf]

Sodium butyrate attenuates microglia-mediated neuroinflammation by modulating the TLR4/MyD88/NF-κB pathway and microbiome-gut-brain axis in cardiac arrest mice

Corresponding author at: Department of Critical Care Medicine, Renmin Hospital of Wuhan University, No. 99 ZhangZhidong Road, Wuhan, Hubei 430060, China. E-mail address: Zhui Yu, yuzhui@whu.edu.cn(Z.Yu).

First author: Jianfei Sun.

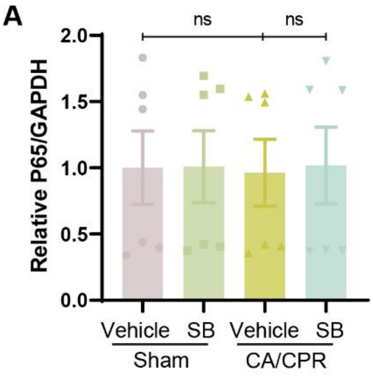

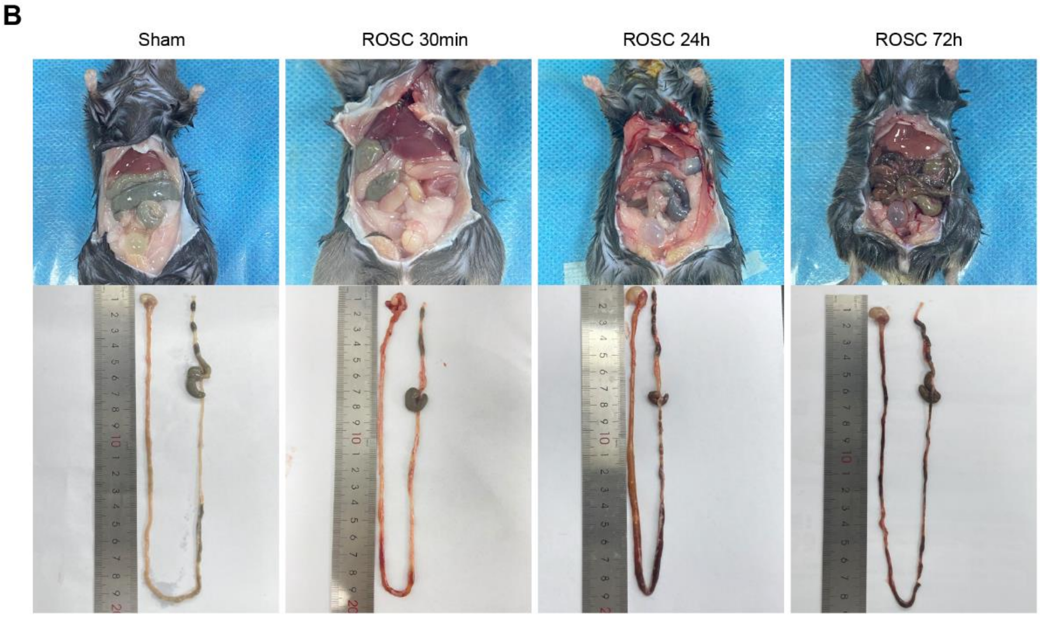

Fig. S1 (A) Quantitative analysis of NF-κB protein expression levels in different groups detected by Western blot. **(B)** Appearance of intestinal tissue in differently treated groups. Data are displayed as mean ±SEM (n=6/group), ns, no significance

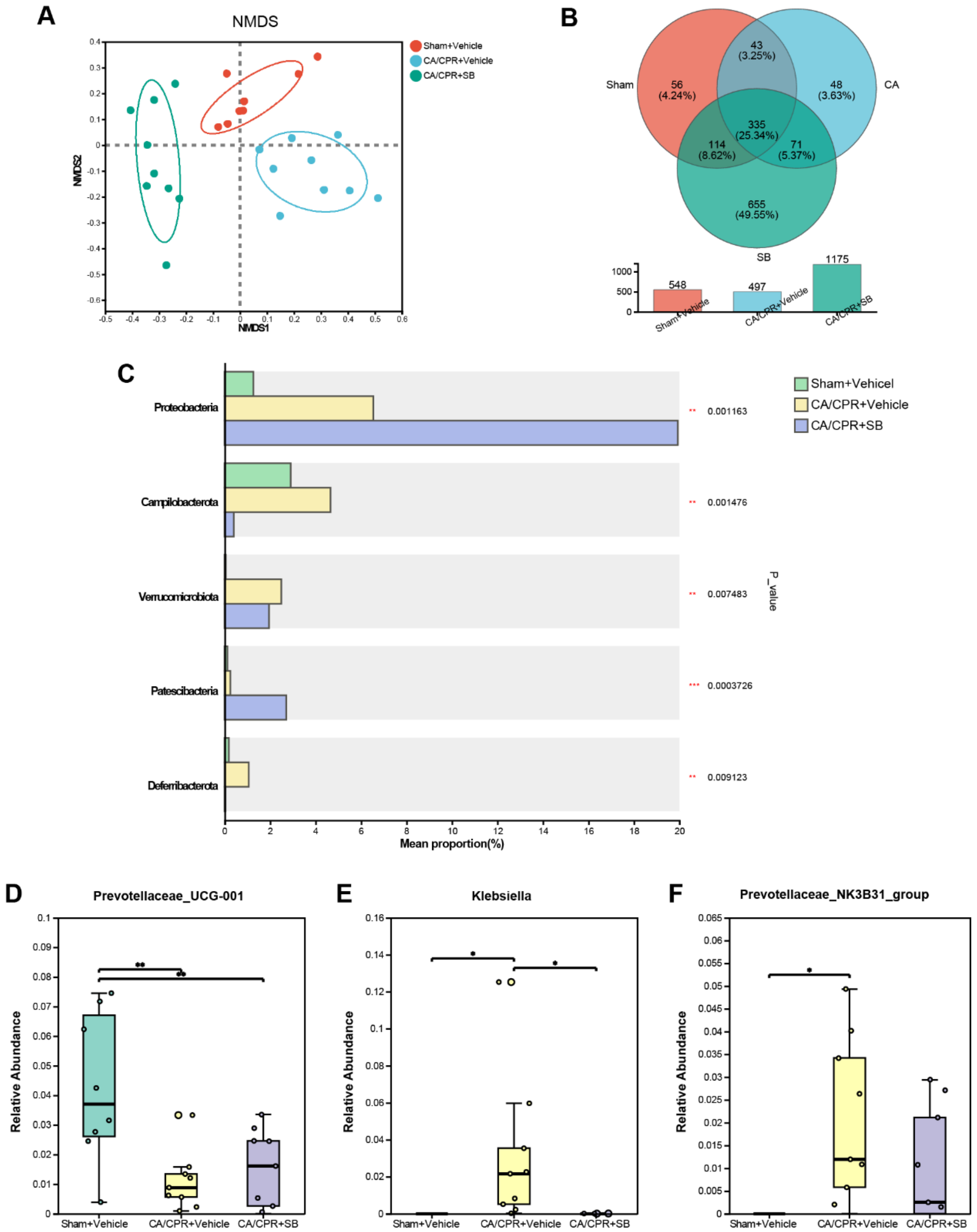

Fig. S2 (A) Beta diversity presented by NMDS. (**B)** The Venn diagram shows OTUs in different groups. (**C**) Differential bacteria at phylum level in the three groups; (**D-F**) The abundance of Prevotellaceae_UCG-001, Klebsiella and Prevotellaceae_NK3B31_group in the three groups. Each boxplot represents the median, interquartile range, and minimum and maximum values. Data were displayed as mean ± SD(n=8-9/group) *$p < 0.05$, **$p < 0.01$, ***$p < 0.001$

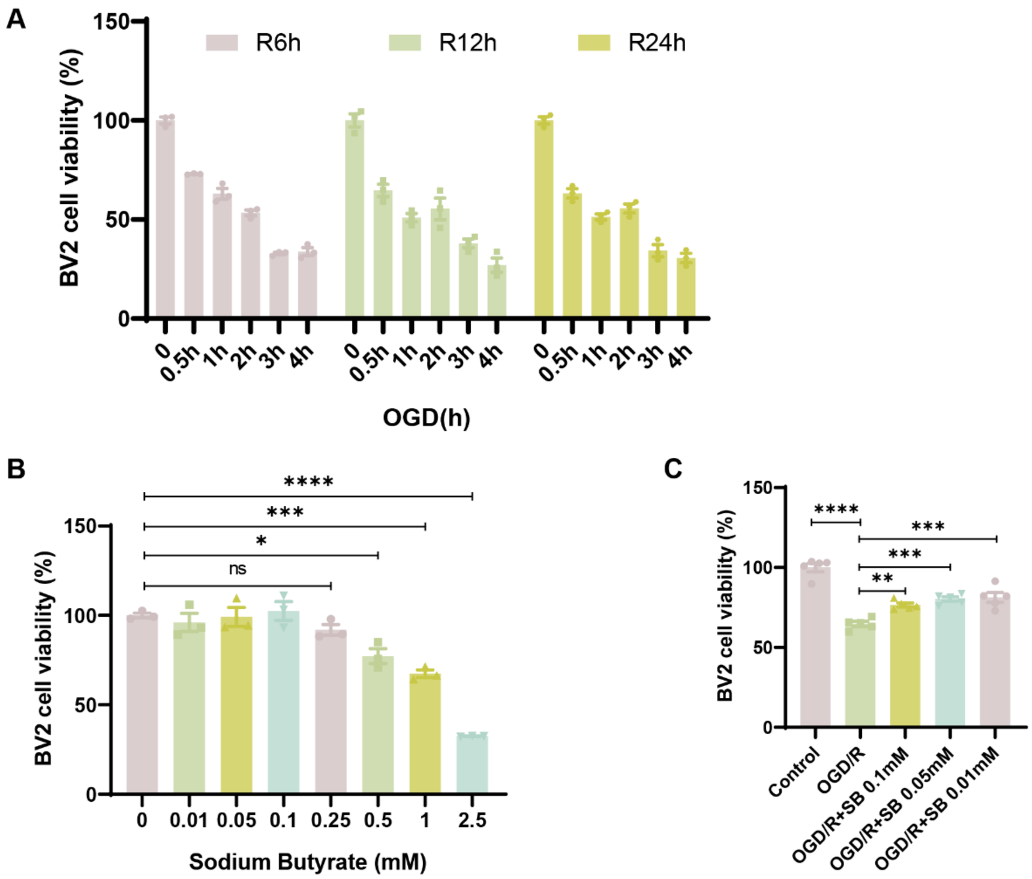

Fig. S3 Cell viability accessed by CCK8 assay. (**A**) OGD/R significantly decreased the viability of BV2 cells. (**B**) SB did not influence the viability of BV2 at doses below 250 μM; however, SB dosages ranging from 0.5 to 2.5 mM dramatically reduced cell viability. (**C**) SB increased the viability of OGD/R-induced BV2 cells. Data are displayed as mean ±SEM (n=5-6/group) *p < 0.05, **p < 0.01, ***p < 0.001, ****p < 0.0001

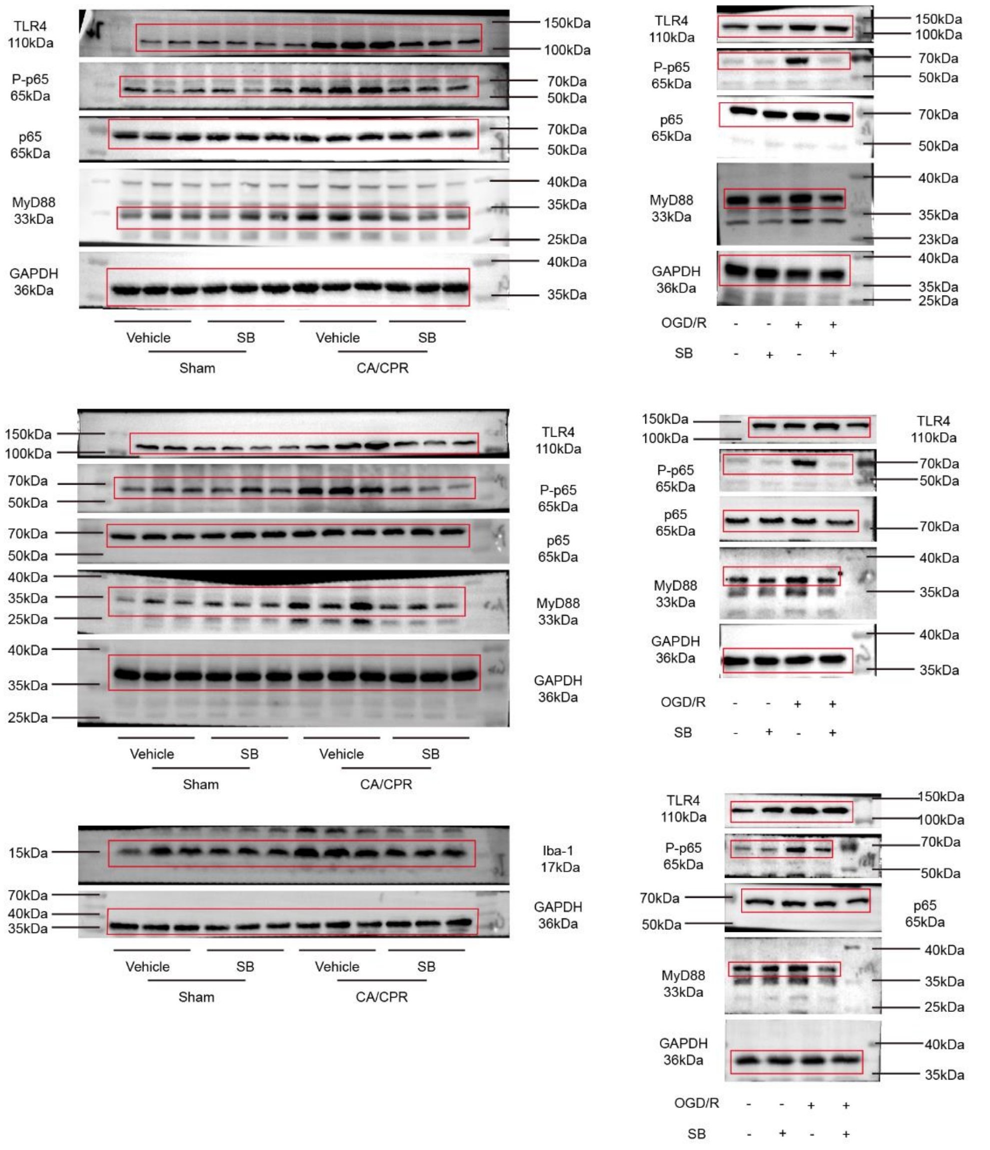

Fig. S4 Original bands of Western blot.
